# Metallochaperones Regulate Intracellular Copper Levels

**DOI:** 10.1371/journal.pcbi.1002880

**Published:** 2013-01-17

**Authors:** W. Lee Pang, Amardeep Kaur, Alexander V. Ratushny, Aleksandar Cvetkovic, Sunil Kumar, Min Pan, Adam P. Arkin, John D. Aitchison, Michael W. W. Adams, Nitin S. Baliga

**Affiliations:** 1 Institute for Systems Biology, Seattle, Washington, United States of America; 2 Seattle Biomedical Research Institute, Seattle, Washington, United States of America; 3 Department of Biochemistry and Molecular Biology, University of Georgia, Athens, Georgia, United States of America; 4 Department of Bioengineering, University of California, Berkeley, California, United States of America; 5 Department of Microbiology, University of Washington, Seattle, Washington, United States of America; University of Wisconsin-Madison, United States of America

## Abstract

Copper (Cu) is an important enzyme co-factor that is also extremely toxic at high intracellular concentrations, making active efflux mechanisms essential for preventing Cu accumulation. Here, we have investigated the mechanistic role of metallochaperones in regulating Cu efflux. We have constructed a computational model of Cu trafficking and efflux based on systems analysis of the Cu stress response of *Halobacterium salinarum*. We have validated several model predictions via assays of transcriptional dynamics and intracellular Cu levels, discovering a completely novel function for metallochaperones. We demonstrate that in addition to trafficking Cu ions, metallochaperones also function as buffers to modulate the transcriptional responsiveness and efficacy of Cu efflux. This buffering function of metallochaperones ultimately sets the upper limit for intracellular Cu levels and provides a mechanistic explanation for previously observed Cu metallochaperone mutation phenotypes.

## Author Summary

Copper (Cu) toxicity is a problem of medical, agricultural, and environmental significance. Cu toxicity severely inhibits growth of plant roots significantly affecting their morphology; Cu overload also accounts for some of the most common metal-metabolism abnormalities and neuropsychiatric problems including Wilson's and Menkes diseases. There is a large body of literature on how Cu enters and exits the cell; the kinetic and structural details of Cu translocation between trafficking, sensing, metabolic, and pumping proteins; and phenotypes associated with defects in metalloregulatory and efflux functions. Although the role of metallochaperones in Cu-cytotoxicity has been poorly studied, it has been observed that in animals deletion of metallochaperones results in elevated intracellular Cu levels along with overexpression of the P1-type ATPase efflux pump, ultimately causing malformation with high mortality. These observations are mechanistically explained by a predictive model of the Cu circuit in *Halobacterium salinarum*, which serves as an excellent model system for Cu trafficking and regulation in organisms with multiple chaperones. Constructed through iterative modeling and experimentation, this model accurately recapitulates known dynamical properties of the Cu circuit and predicts that intracellular Cu-buffering emerges as a consequence of the interplay of paralogous metallochaperones that traffic and allocate Cu to distinct targets.

## Introduction

Copper (Cu) is an essential trace element in nearly all biological systems [1] but highly cytotoxic when in excess [2], [3]. Biological systems possess sophisticated trafficking systems that include ion importers to control Cu entry [4]; metallochaperones for shuttling intracellular ions to and from targets[5], [6]; efflux pumps that export excess Cu [7], [8]; and metalloregulators that sense internal abundance and modulate expression of all trafficking proteins [9]. There is a large body of literature on how Cu enters and exits the cell [10], [11]; the kinetic and structural details of Cu translocation between trafficking, sensing, metabolic, and pumping proteins [12], [13], [14]; and phenotypes associated with defects in metalloregulatory and efflux functions [15].

Defects in Cu efflux pumps, for example, are associated with neurodegenerative disorders such as Menkes and Wilson's syndromes, and Alzheimer's disease [7], [16], [17], [18], [19]. Although we do not fully understand the mechanistic role of metallochaperones in any Cu-related disease, they are universally present [20], [21], [22], [23], [24], [25], [26] and known to be important in Cu-trafficking and preventing cellular damage [27], [28]. Furthermore, deletion of the Cu metallochaperone Atox1 in immortalized human cell lines resulted in elevated intracellular Cu levels along with overexpression of Cu transporting ATP7A [19], [29]. Mice harboring metallochaperone deletions are malformed with high mortality [30]. Combined, these data suggest that beyond their known Cu trafficking role, metallochaperones also influence the activity of metalloregulators and intracellular Cu levels via an uncharacterized mechanism.

In this study we present an integrated experimental and computational analysis of transcriptional regulation of Cu efflux in *Halobacterium salinarum NRC-1* and the role that metallochaperones play therein. In brief, *H. salinarum* possesses a post-transcriptionally regulated Cu trafficking system that was characterized by systems analysis of cellular response to growth sub-inhibitory levels of extracellular Cu. The main components of the Cu efflux network in *H. salinarum* include a Cu sensing metalloregulator (VNG1179C) that regulates transcription of a P1-type ATPase efflux pump (VNG0700G), and two metallochaperones (VNG0702H and VNG2581H) with Cu binding sites that are highly conserved across all domains of life (Figure S1). We integrated the functional interplay between these components into a system of ordinary differential equations with iterative refinement of model parameters to fit experimental data of Cu response dynamics. Using this model we explored the consequence of deleting or overexpressing metallochaperones on activity of VNG1179C (assayed directly by measuring VNG0700G transcript levels, or indirectly by measuring fluorescence in live cells transformed with a GFP reporter tagged to the promoter of VNG0700G) and ultimately on intracellular Cu levels (measured with ICP-MS). The three rounds of iterative experimentation and computation has revealed that each of the two metallochaperones in *H. salinarum* have distinct functions, and that their interactions with other components of Cu efflux acts as a buffer, setting the upper threshold of homeostatic intracellular Cu. Altering the absolute abundance of metallochaperones significantly affects sensitivity of the metalloregulator to Cu levels, efficacy of Cu efflux by VNG0700G, and ultimately results in higher level of intracellular Cu.

## Materials and Methods

### Cell culture

Glycerol stocks of *H. salinarum* with requisite gene knockouts were revived on solid CM (NaCl–250 g/l, MgSO_4_•7H_2_O–20 g/l, Na·Citrate–3 g/l, KCl-2 g/l, and peptone 10 g/l) agar (1.8% w/v) plates incubated at 37°C for 1–2 wks. Single colonies were selected from plates and placed into liquid CM media (typically 50 ml in a 125 ml Erlenmeyer flask unless otherwise noted) and grown to an optical density (measured at 600 nm) (OD600) of 0.6–0.8 to create stock cultures. Prior to experiments, stock cultures were split into replicates and diluted in fresh medium to a starting OD600 of 0.05. Cu addition experiments were initiated once replicate cultures reached OD600 of 0.4–0.6. Strains carrying GFP expression vectors required medium supplemented with 200 ng/ul mevinolin.

### Strain construction

Mutant strains harboring deletions of *VNG1179C*, *VNG0702H*, and *VNG2581H* were created via a two step in-frame deletion method previously described [31], [32] using a *Δura3* strain as the parent background.

### mRNA profiling via Microarray analysis

Strains studied were grown in liquid cultures 250 ml in volume (500 ml flask) at 37°C to accommodate 4 ml samples taken at specified timepoints. Total RNA was purified from cell pellet lysates, stained, and hybridized to a spotted microarray using a previously reported dye-flip protocol [31]. Transcriptome expression data presented herein is original to this work.

### ICP-MS analysis

Metal free basal salts solution (NaCl–250 g/l, MgSO_4_•7H_2_O–20 g/l, Na·Citrate–3 g/l, KCl-2 g/l) and MilliQ water was created by overnight treatment with 5 g/l Chelex. For metal free basal salts solution, metal pure MgSO_4_ (<0.001% metal impurity) was added after Chelex treatment to avoid saturating the ion binding capacity of the resin. Prior to experiments, all glassware and sample collection tubes were washed twice in 2% nitric acid to strip any trace metals. For ICP-MS analysis, 10 ml samples were retrieved from cultures pre- and post- copper addition into 50 ml conical tubes. Cells were pelleted by centrifugation and washed three times in basal salts solution. After the final wash, cells were lysed in MilliQ water and sonicated for 10 min and stored at 4°C for analysis. Samples were analyzed by ICP-MS using minor modifications to previously published protocols [33].

### GFP assays via flow cytometry

GFP timeseries experiments were conducted using 50 ml cultures seeded with clonal populations of cells from solid medium colonies. Once cultures reached an OD600 of 0.4–0.6, CuSO_4_•5H_2_O was spiked in to a final concentration of 0.85 mM. At selected timepoints, 500 ul samples were taken from cultures and pelleted. Cells were then fixed by resuspending in 1 ml of basal salts solution with 0.25% (w/v) paraformaldehyde and incubated at room temp for 10 min. Fixative was removed by pelleting cells and resuspending in basal salts solution and samples were stored at 4°C.

GFP Cu dose experiments were conducted in 96 deep-well plates (1.6 ml culture per well). Cultures were started in 500 ml flasks and distributed to wells at a nominal OD600 of 0.05. Plates were sealed with BreathEasy film and incubated at 37°C in a shaking incubator set to 200 rpm. Once the average OD600 of the plate reached 0.4–0.6, a pre- Cu addition sample was taken and processed as below. Afterwards, CuSO_4_•5H_2_O was spiked in and the plate was incubated for 300 min at 37°C, shaking at 200 rpm, at which time an endpoint sample was taken. For each sample, 150 ul of culture was removed from each well and transferred to a v-bottomed 96-well plate prealiquoted with basal salts solution with 0.5% (w/v) paraformaldehyde. Cells were pelleted in a tabletop centrifuge at 2800G for 10 min at room temp. Supernatant fixative was removed and cells were resuspended in basal salts solution. Plates were then sealed with aluminum foil tape stored at 4°C until analysis.

Flow cytometry analysis was performed within 2–3days of sample collection using a BD InFlux cell sorter fitted with a 100 um flow nozzle and primed with 100 g/l NaCl in MilliQ water as sheath. Prior to injection, each sample was spiked with 1 um yellow/green fluorescent beads to a final concentration of 1×10^7^ ml^−1^ for use as an internal reference. 100,000 total events were collected and population gating was done via FlowJo. Gated cell populations were exported to ASCII files and further analyzed using the statistical analysis environment, R (http://www.r-project.org).

In R, fluorescence concentrations were calculated by dividing each event's GFP signal by its corresponding forward scatter value. Population concentration means resulting from this analysis are presented in the results.

### Model details

#### Equations

The model is formulated as a set of discrete mass-action chemical reactions that take into account known and predicted interactions among the various components of the Cu-efflux regulatory circuit.

Cu-responsive transcription via the Cu-sensing metalloregulator P1179 for chromosomal genes is given by:
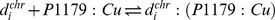
(1)

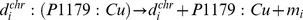
(2)where 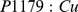 is the Cu activated form of P1179, 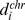 is the chromosomal gene copy, and 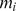 is the transcript for species 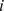. Reaction eq. (1) is reversible and is determined by two rate parameters for the forward (k.Di.P1179.Cu.binding) and reverse (k.Di.P1179.Cu.dissocation) reactions. Reaction eq. (2) specifies that at transcription termination, the transcriptional complex of DNA, regulator, and transcript completely decouple.

When necessary, constitutive transcript overexpression of species 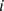 via plasmid DNA (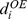) occurs via the reaction:
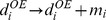
(3)Notice that no active regulator is required to produce transcript from 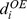.

Transcripts 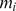 produced by reaction eqns. (1), (2), and (3) are translated to proteins 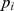 by:
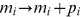
(4)where ribosome binding has been excluded for simplicity.

Both transcripts and proteins undergo degradation reactions:
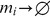
(5)

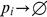
(6)with the exception of regulator proteins P1179.

These transcription and translation reactions were included for dynamically expressed species – YvgX, Chaperones, and GFP. Importantly, explicitly describing these dynamics (as opposed to using lumped or “aggregate” rate laws), allows for further corroboration of model predictions to experimental data – i.e. temporal changes in transcript level expression profiles assayed via microarray.

The Cu sensitive metalloregulator P1179 is both activated and deactivated by Cu via metallochaperone (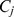 = P0702 or P2581) mediated trafficking:
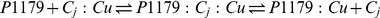
(7)where reaction intermediates of the form 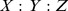 are equivalent to 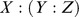 and 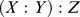, such that:
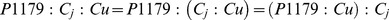
(8)represents the inter-protein transfer of Cu ions to/from the metallochaperone 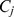 from/to the regulator 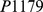.

In addition, P1179 may acquire/lose Cu via non-specific binding/debinding:
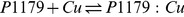
(9)


These non-specific reactions occur at a low rate to account for the P1179's need for metallochaperones to efficiently bind Cu, and the regulator's high affinity for Cu once bound.

Metallochaperones acquire Cu via bulk adsorption:
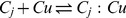
(10)and chaperone-chaperone ion transfers (multi-metallochaperone models 1.x):
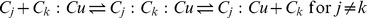
(11)As with the reverse reaction in eq. (9), it is assumed that metallochaperones have a high affinity for Cu, thus the reverse rate for reaction eq. (10) is similarly small.

Quota elements are loaded irreversibly with Cu via metallochaperones and non-specific adsorption:
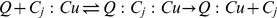
(12)

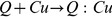
(13)


Cellular growth and associated maintenance occurs via the following reaction:
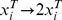
(14)where 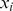 is DNA (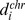, 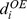), Cu quota elements (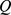), cell volume (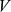), and metalloregulator P1179. The superscript ^T^ indicates total abundance within the cell such that:
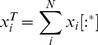
(15)where [:*] indicates any other binding partners for 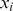. For example:
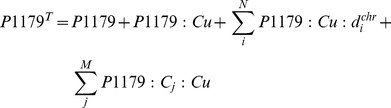
(16)


These equations produce fresh pools of apo-/uncomplexed species and also establish a constant ratio of “grown” species to the cell volume to avoid unwanted dilution effects.

Cu import was modeled as a simple linear gradient, with partitioning at the cell membrane:
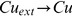
(17)where the reaction rate was defined as:
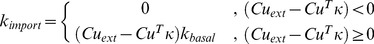
(18)for all copper ions in the cell 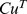, membrane partition coefficient 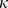, and basal rate 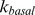. Here it was assumed that Cu could only irreversibly cross the cell membrane via an unspecified Cu specific importer/ion pore.

Cu export was assumed to be only mediated by the exporter YvgX (P0700):
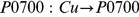
(19)with copper loading of the pump occurring via non-specific and chaperone mediated mechanisms:
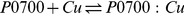
(20)

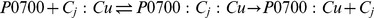
(21)


Notably, we have explicitly defined trafficking interactions of Cu as this is a key component of metallochaperone function and a focal point for experimentally testing model predictions.

#### Simulation

The system of reactions was simulated numerically using the following differential equation:
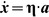
(22)where 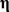 is a stoichiometry coefficient matrix mapping reactants (rows) to reactions (columns) and 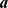 is a propensity/reaction rate vector with elements of the form:
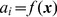
(23)and is typically:
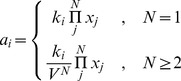
(24)


Importantly, multi-molecular reactions where the number of reactants is ≥2 are adjusted by the cell volume V to account for increased diffusional resistance [34], [35].

Note, for bidirectional reactions, both forward and reverse reactions are defined explicitly. The stoichiometric sign in 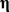 defines the direction of flux for each species pool 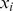 and reaction propensities 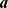 are 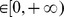.

While slightly complicated, the method by which the model is formulated makes it easy to extend without having to rederive analytical forms of ODEs. In fact, all models used in this paper are derived from one large “master model” where specific reactions are deactivated as necessary by setting their respective rates 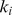 to 0.

#### Parameters

The full parameter set is available via the model source code. Specific assumptions made in the derivation of parameters are detailed below.

Rates for dynamically expressed species (YvgX and metallochaperones – VNG0702H, and VNG2581H) were determined via fits of timeseries microarray (mRNA transcription and degradation) and GFP (protein translation) expression data. Protein degradation was assumed to be dominated by dilution and therefore had a rate of 0 unless otherwise specified. These fits were used to extrapolate a generic RNA polymerase production rate to estimate the transcription rate of GFP based solely on gene length and a ribosomal production rate used to estimate translation of based on resultant peptide length.

Cellular growth was assumed to have a doubling time of 7 h, consistent with growth rates observed during exponential growth of *H. salinarum*.

Since precise rates for Cu binding events were unknown, the following assumptions were made. Direct Cu binding by metallochaperones were assumed to be on the order of protein translation (0.01 events/s, rates are here-on reported as events/s unless otherwise specified). The first step in metallochaperone mediated Cu transfers was also set to 0.01. The rate of Cu-bound chaperone and target dissociation without Cu transfer was set to 0.1, while dissociation with transfer was set to 1. In combination, these Cu binding parameters create a net forward flux from free Cu and apo-chaperones to Cu-bound chaperons.

The rate of non-specific Cu binding for all non-chaperone Cu binding species was set to be on the order of protein degradation (0.001). The rate of spontaneous debinding of Cu from metallochaperones was also set to this rate. Cu efflux and Cu sensing regulatory proteins (VNG1179C) were assumed to have a high Cu affinity, thus spontaneous Cu dissociation from these species was set to 0.0001, 10-fold less than protein degradation. Thus, the primary mechanism by which any Cu bound species could be relieved of Cu was by chaperone mediated trafficking.

The protein abundance of VNG1179C is held constant relative to cell volume via eq. (16) such that each dynamically regulated gene has nominally two copies of regulator available.

The rate of constitutive expression of the metallochaperones via eq. (3) was held constant and but scaled by 100-fold relative to VNG1179C mediated transcription to account for inherent delays from transcription factor binding.

Specific modifications to the parameter set made by the various models are summarized in Table 1.

**Table 1:** Abridged parameter set.

Parameter	Default	Model 0	Model 1.1	Model 1.2	Model 1.3
k.D2581.P1179.Cu.binding	0.01	0			
k.D2581.P1179.Cu.dissociation	0.1	0			
k.M2581.degradation	0.003262	0			0.001367
k.M2581.transcription	0.424167	0			0.646571
k.OE2581.transcription	0.004242	0			0.006466
k.P0700.Cu.by.P2581.F1	0.01	0		0	0.001
k.P0700.Cu.by.P2581.R1	0.1	0		0	
k.P0700.Cu.by.P2581.F2	1	0	0.1	0	
k.P0702.Cu.bind	0.01				0.0002
k.P0702.Cu.by.P1179.F1	0.01			0	0
k.P0702.Cu.by.Q.F1	0.001			0	0
k.P0702.Cu.by.P2581.F1	0.01	0	0		
k.P0702.Cu.by.P2581.R1	0.1	0	0		
k.P0702.degradation	0				0.0002
k.P2581.Cu.bind	0.01	0			
k.P2581.Cu.by.P1179.F1	0.01	0			
k.P2581.Cu.by.P0702.F1	0.01	0	0		
k.P2581.Cu.by.P0702.R1	0.1	0	0		
k.P2581.Cu.by.Q.F1	0.001	0			
k.P2581.Cu.debind	0.001	0			
k.P2581.Cu.degradation	0	0			
k.P2581.degradation	0	0			0.0005
k.P2581.translation	0.030769	0			0.028986
k.P1179.Cu.by.P0702.F1	0.01			0	0
k.P1179.Cu.by.P0702.R1	0.1			0	0
k.P1179.Cu.by.P0702.F2	0.1			0	0
k.P1179.Cu.by.P2581.F1	0.01	0			
k.P1179.Cu.by.P2581.R1	0.1	0			0.01
k.P1179.Cu.by.P2581.F2	0.1	0	0.01		0.05
k.Q.Cu.by.P0702.F1	0.01			0	0

All rates listed above are in units of events/s unless otherwise specified.

#### Initial conditions

All species within the cell had 0 initial quantities except the following. Quota elements (*Q*) were initialized to 1000 molecules/cell. The genome copy number was set to 15. Regulatory protein (P1179) abundance was set to 6× the genome copy. The initial cell volume was 1. In knockout simulations, genome copy was set to zero for appropriate DNA abundances.

#### Model simulations and code

Computational simulations were performed using the statistical analysis platform R and the ODE simulation package *deSolve*. Model source code is available for download via GitHub (http://github.com/wleepang/CuEfflux). Also included is code to convert the R based model code to SBML.

## Results

### Transcriptional control of metallochaperones

**Figure 1:**
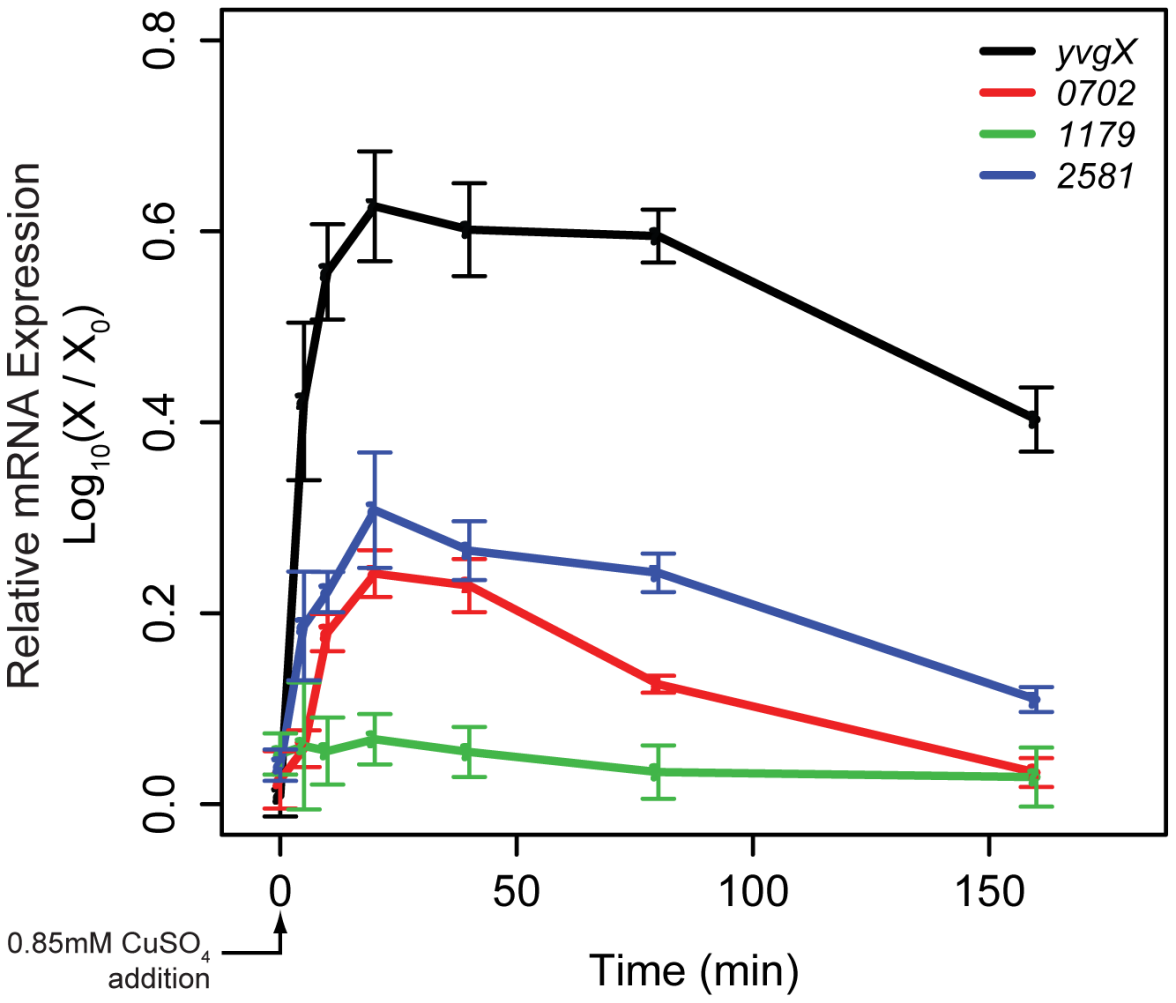
Transcriptional response of components of the Cu efflux network and phenotypic consequences of their deletion. Temporal changes in mRNA levels in response to a step increase in Cu to growth sub-inhibitory level (0.85 mM) reveals pulsed responses in transcript expression of the efflux pump *yvgX* and the two metallochaperones *VNG0702H* and *VNG2581H*. Expression of the metalloregulator *VNG1179C* does not change, suggesting that it is post-transcriptionally activated. Error bars are s.e.m for n = 5. Points (X) within each expression profile were normalized to the expression value at t = 0 (X_0_). This ratio is plotted using a Log_10_ transformation to accommodate large differences in dynamic range. Negative values indicate decreases in expression relative to expression at t = 0.

When stressed with excess Cu, activation of efflux mechanisms protects the cell by restoring intracellular Cu to homeostatic levels. To determine the temporal dynamics of transcription of all genes involved in Cu efflux, we performed a global time course survey of transcript level changes in cells stressed with 0.85 mM CuSO_4_, a previously determined growth sub-inhibitory Cu concentration [31]. To rid the cell of excess Cu, we discovered that *H. salinarum* relies primarily on transcriptional activation of three genes: a P1-type ATPase efflux pump *yvgX* (*VNG0700G*), and two HMA-domain containing metallochaperones *VNG0702H* and *VNG2581H*. The temporal transcript changes for each of these genes demonstrated pulsed induction following Cu exposure (Figure 1). These genes were also previously determined to be regulated by *VNG1179C* [31], a Lrp family transcription factor with a TRASH domain for metal sensing [36]. The expression of this regulator did not change under Cu stress suggesting that it is post-translationally activated – e.g. upon binding Cu.

The pulse-like transcriptional response of *yvgX* was expected due to its direct role in relieving the cell of excess intracellular Cu. Conversely, the pulse in metallochaperone expression was unexpected. Absent information on transcriptional dynamics of metallochaperones, we had initially assumed that they were constitutively expressed at low levels. However, the regulated transcription of these genes in direct response to excess Cu suggested that the metallochaperones might have an important role in tuning the transcriptional dynamics of the Cu efflux network. Notably, negative feedback linking activity of YvgX to repression of VNG1179C must exist to conserve cellular resources. The mechanisms that enable such feedback are most likely indirect as YvgX is membrane bound and VNG1179C is cytoplasmic and bound to DNA. Our hypothesis was that feedback must occur via metallochaperones because of their ability to interact directly with Cu ions, VNG1179C, and YvgX.

### Metallochaperones modulate responsiveness of the metalloregulator and, thereby, maintain intracellular Cu level within the homeostatic range

**Figure 2:**
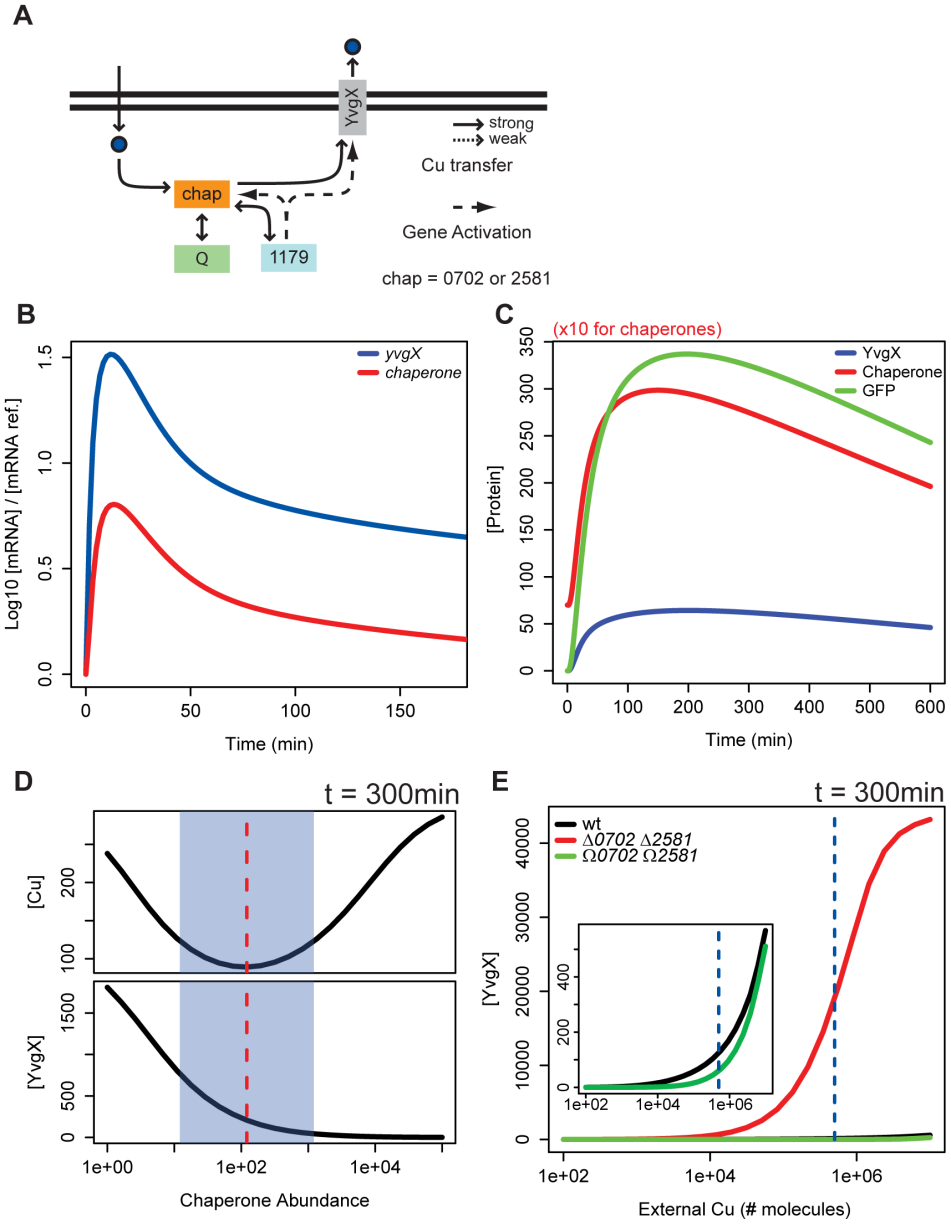
A model for the role of metallochaperones in modulating transcriptional dynamics and intracellular Cu levels. (A) Schematic of the Cu efflux network with a single metallochaperone (Model 0). At the core is the interaction of the metallochaperones (yellow) with all known elements of the Cu efflux network – Cu ions (dark blue), efflux pump (gray), metalloregulator (light blue), and intracellular ion quota (scavenging/metabolizing enzymes) (green). (B) Model 0 recapitulates pulsed transcriptional induction of the efflux pump and metallochaperone. Simulated mRNA expression profiles for *yvgX*, metallochaperones, and *gfp*. (C) Simulated protein expression profiles for YvgX, metallochaperones, and GFP. Metallochaperone levels have been reduced by a factor of 10 for plotting. (D) Model 0 predicts that changes in metallochaperone abundance have significant consequences on steady state levels of YvgX and intracellular Cu. Simulated scan of steady state level for YvgX expression and intracellular Cu relative to metallochaperone abundance. Shaded region is the optimum range for metallochaperone abundance and corresponds to the levels observed in simulations of Cu efflux in a *wt* background. (E) Simulated scan of steady state level for YvgX expression in cells with normal (black), deficient (red), and high (green) metallochaperone expression levels. Blue dashed line indicates 5×10^5^ molecules of external Cu concentration in both simulations and experiments.

Based on existing mechanistic understanding from diverse organisms [11], [37], [38], our prior work [31], and the dynamics of the transcriptional response investigated in this study, we developed a computational model for the transcriptional regulation of Cu efflux (Figure 2A). A system of ordinary differential equations that describe transcriptional, translational, and post-translational regulatory events, this model (Model 0) makes two important assumptions based on known biology. First, intracellular Cu is rarely unchelated or “free” [25], but instead is readily bound by metallothioneins [9], [13], [39], glutathione [40], [41], and other Cu binding proteins [2]. We modeled this Cu sequestration capacity with a quota element (*Q*) whose demand is fulfilled prior to activation of Cu efflux. Second, we assumed that the two metallochaperones VNG0702H and VNG2581H were functionally indistinguishable given the high level of similarity in both their expression profiles and Cu binding motifs. Therefore, metallochaperones were simulated as a single species with two-fold higher copy number than other elements.

We tested this model by performing simulations of the Cu response to a step-increase in extracellular Cu to a growth sub-inhibitory level (0.85 mM). The model assumes that a single cell only reacts to a shell of surrounding volume equivalent to 3× the cell volume. A concentration of 0.85 mM of dissolved copper therein is equivalent to ∼5×10^5^ molecules. This value is held constant throughout simulations, representing non-changing copper supply in the bulk growth medium and presenting a boundary condition for copper at the cell membrane.

All simulations were run until 300 min to give sufficient time for restoration of regulated Cu homeostasis in normal (wild-type or *wt*) cells. The simulations accurately recapitulated known dynamics of transcriptional induction of *yvgX* and metallochaperones with transcript and protein levels peaking at ∼18 and 200 min, respectively (Figures 2B and 2C). Based on these encouraging results we proceeded to explore the functional and mechanistic role of metallochaperones in regulation of Cu efflux.

First, we investigated whether changes in abundance of metallochaperones had any consequence on expression of *yvgX* and, ultimately, on intracellular Cu concentration. The model predicted that the metallochaperones had to be within an optimal range of 100–1000 molecules per cell to produce 100–200 copies of YvgX for maintaining low levels of intracellular Cu (Figure 2D). Interestingly, increasing or decreasing the concentration of metallochaperones outside this optimal range had significantly different effects on steady state levels of YvgX and intracellular Cu. Lowering the abundance of metallochaperones below 100 molecules per cell resulted in increased levels of both intracellular Cu and YvgX. In contrast, increasing the metallochaperones to above 1000 molecules per cell resulted in Cu accumulation with undetectable YvgX levels.

Next, we investigated the consequence of increasing or decreasing metallochaperone abundance on sensitivity of the Cu efflux response over a wide range of extracellular Cu concentrations. The model predicted significant differences in the threshold concentrations of Cu that were necessary to activate expression of YvgX in presence of low, normal, and high abundance of metallochaperone (Figure 2E). Depletion of metallochaperones was predicted to significantly increase the sensitivity of the response, with steady state YvgX levels rising to 1000 copies per cell in the presence of micromolar quantities of external Cu. In contrast, overexpression of metallochaperones was predicted to repress YvgX expression over almost the entire range of extracellular Cu. Thus, the model predicted that metallochaperones tune responsiveness of the metalloregulator, modulate the absolute abundance of the efflux pump, and, ultimately, set the homeostatic level of Cu.

#### Experimental verification

**Figure 3:**
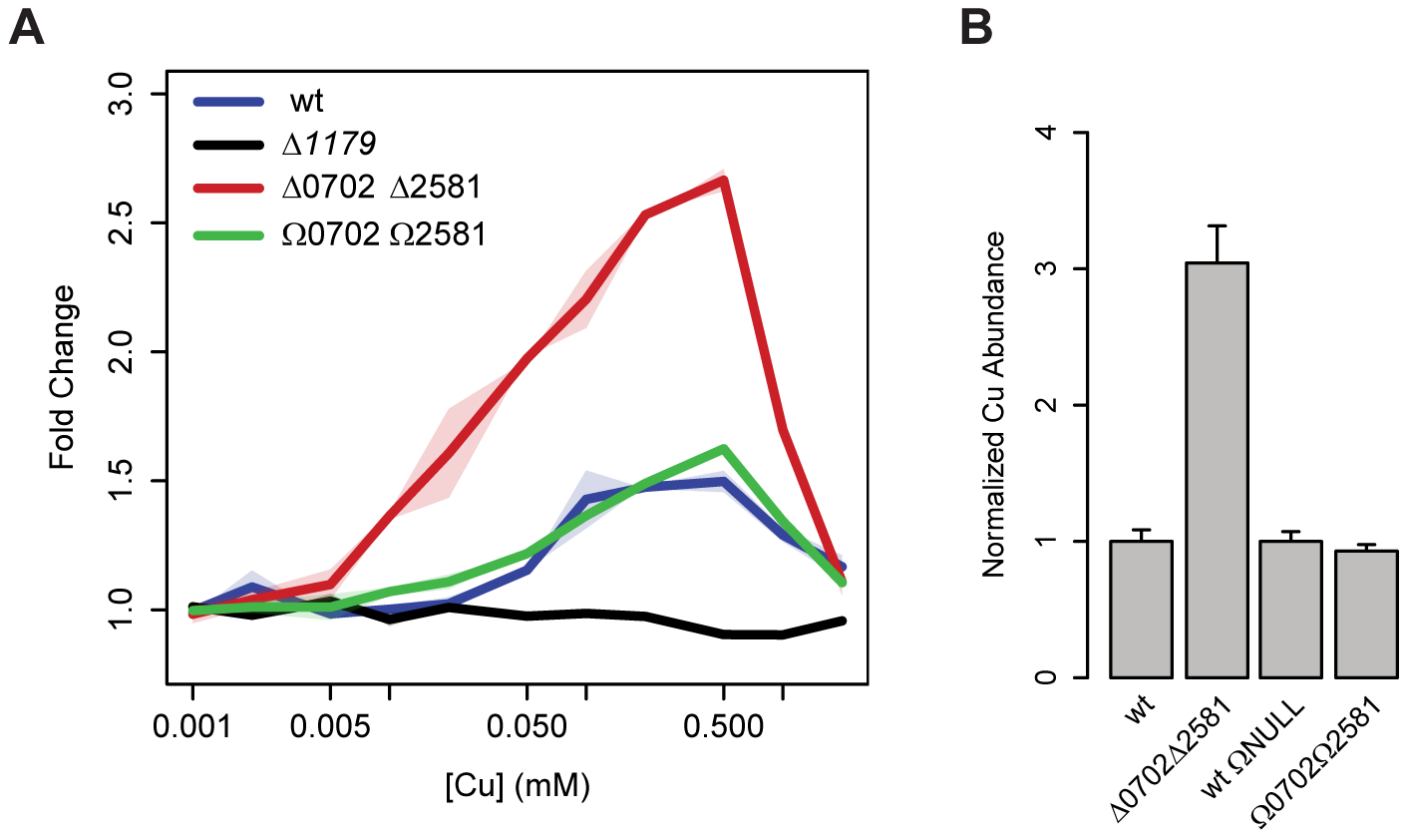
Transcriptional dynamics of YvgX and associated consequences on intracellular Cu levels. (A) Experimental validation of perturbed *yvgX* activation sensitivity in cells with normal (*wt*, blue), deficient (*Δ0702Δ2581*, red), and increased (*Ω0702Ω2581*, green) metallochaperone levels, measured at 300 min post challenge with increasing concentrations of CuSO_4_. Solid lines are averages of two independent biological replicates. Shaded areas show the spread between replicates. (B) Experimental validation of perturbed intracellular Cu concentrations in cells with normal, deficient, and increased metallochaperone levels after 300 min post stimulus with 0.85 mM CuSO_4_ were measured using inductively coupled plasma mass spectrometry (ICP-MS).

Model 0 predicted several important outcomes of changing the abundance of metallochaperones that could be directly tested by measuring changes in *yvgX* transcript levels and intracellular Cu. To simultaneously monitor the activity of the metalloregulator and the expression levels of *yvgX*, we transcriptionally fused GFP to the *yvgX* promoter on a low copy number plasmid (pLP17, Figure S2). Along with the *wt* (*Δura3*) background, we introduced this promoter-GFP system into strains with deletion of the metalloregulator (*Δ1179*), deletion of both metallochaperones (*Δ0702Δ2581*), and constitutive overexpression of both metallochaperones (*Ω0702Ω2581*, pLP20, Figure S3). The transcriptional fusion to GFP enabled non-invasive flow cytometry-based fluorescence measurements as a proxy for tracking intracellular *yvgX* transcript level changes in all of these strains. We first confirmed that when stressed with growth sub-inhibitory Cu, fluorescence changes due to GFP synthesis and turnover accurately recapitulated the temporal induction of *yvgX* transcription in a *wt* background (Figure S4). Second, we also ascertained that there was no Cu-mediated induction of *yvgX* in the metalloregulator knockout background (*Δ1179*) (Figures 3A and S4), demonstrating that the promoter-GFP fusion essentially reports on the activity of the metalloregulator.

We then proceeded to test model predictions by assaying the consequences of deleting or overexpressing the two chaperones on transcriptional induction of *yvgX* under Cu stress (Text S1 and Figures S5 and S6). Consistent with model predictions (Figure 2E), deletion of both chaperones (*Δ0702Δ2581* strain) significantly increased the sensitivity of the metalloregulator to small increases in extracellular Cu. In contrast, *yvgX* transcript level in the metallochaperone overexpression background (*Ω0702Ω2581*) was identical to that in the *wt* (Figure 3A).

To measure intracellular Cu, we used inductively coupled plasma mass spectrometry (ICP-MS), a sensitive method for interrogating metals in biological samples [33]. In agreement with model predictions *Δ0702Δ2581* had elevated Cu levels 300 min after a step increase in extracellular Cu to 0.85 mM. In contrast, *Ω0702Ω2581* did not significantly accumulate Cu relative to *wt* (Figure 3B).

In summary, experimental validations of predictions made by the simplified model of Cu efflux corroborates that depletion of metallochaperones significantly increases transcription of *yvgX* and intracellular accumulation of Cu. By validating model predictions we have demonstrated that the abundance of metallochaperones is tuned to keep intracellular Cu in a range that meets the biological needs of the cell and below a level that is toxic. In other words, metallochaperones function as a transcriptionally-tuned buffering system for Cu. However, discrepancy in predicted and experimentally measured intracellular Cu levels when metallochaperones are overexpressed demanded revisions to the model through incorporation of additional mechanistic detail(s). Since Model 0 accurately reproduced known *yvgX* expression dynamics, we focused our revision efforts on tuning the model to resolve discrepancies in intracellular Cu concentrations.

### Extended model predicts different functions for VNG0702 and VNG2581

In Model 0 we made the assumption that both metallochaperones had identical functions and, therefore, provided equivalent Cu trafficking and buffering capacity. Alternatively, there could be differences in the specific roles of each metallochaperone. Eukaryotes make use of several Cu-specific metallochaperones: ATOX1, CCS1, and COX17. ATOX1, the eukaryotic ortholog of metallochaperones in *H. salinarum*, trafficks Cu to P1-type ATPases ATP7A and ATP7B [29] while CCS1 delivers Cu to Cu/Zn dismutases and COX17 delivers Cu to cytochrome oxidases [27]. This raises the possibility that there might be similar distinction in Cu trafficking by the two metallochaperones in *H. salinarum*.

**Figure 4:**
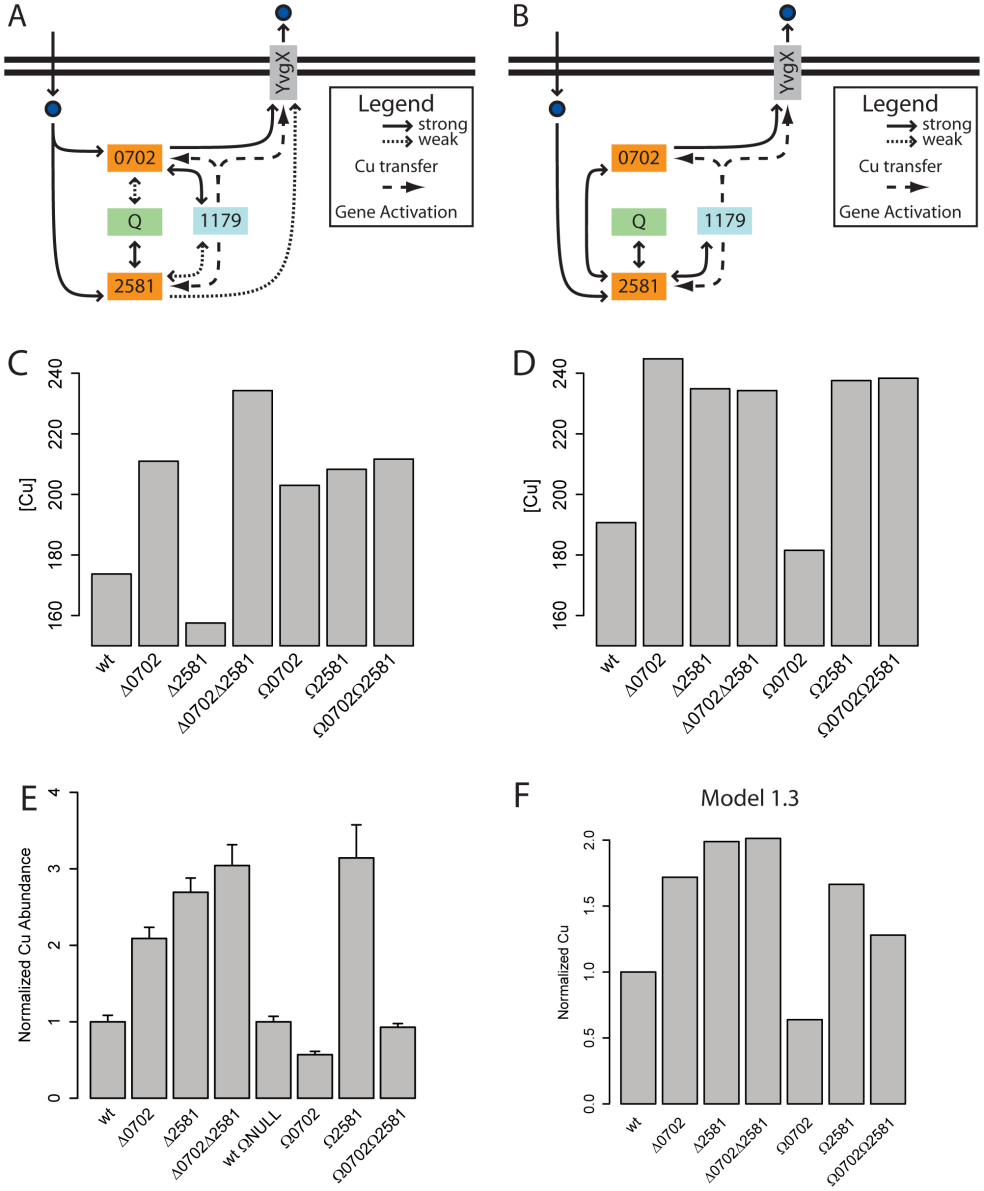
Model for Cu trafficking, transcriptional regulation and efflux with nuanced and distinct functions for the two metallochaperone paralogs. (A) Network for a model which assumes that the two metallochaperones have equivalent functions with nuances in their primary and secondary targets (Model 1.1). The delivery of Cu to primary targets is assumed to be “strong” or preferred but Cu hand-off to secondary targets also occurs albeit through “weaker” interactions. (B) Network for a model which assumes VNG0702H and VNG2581H have distinct roles in Cu trafficking (Model 1.2). Whereas Cu efflux is mediated only by VNG0702H, other trafficking roles are performed by VNG2581H. Notably, VNG0702H must receive Cu via a transfer from VNG2581H, which directly binds imported Cu. (C) Model 1.1 predictions do not recapitulate the experimental data – e.g. incorrect predictions of intracellular Cu levels in three of the four metallochaperones perturbed genetic backgrounds, i.e. *Δ2581*, *Ω0702* and *Ω0702Ω2581H*. (D) Accurate predictions of Cu accumulation in all strains (with the exception of *Ω0702Ω2581*) by Model 1.2 support distinct roles for the two metallochaperones. Specifically, the model predicts relative intracellular Cu levels across all strains to be in the following order: *Δ0702>Δ2581 = Δ0702Δ2581 = Ω2581 = Ω0702Ω2581>wt>Ω0702*. (**E**) ICP-MS measured intracellular Cu concentrations after 300 min for single deletion and overexpression of metallochaperones. Double deletion and overexpression data are shown again for comparison. Here intracellular Cu levels nearly match Model 1.2 predictions: *Δ0702Δ2581>Δ2581>Δ0702 = Ω2581>Ω0702Ω2581 = wt>Ω0702*. (**F**) Predictions of intracellular Cu concentrations after 300 min by Model 1.3 (whose topology was determined by steady-state analysis to be a hybrid of Models 1.1 and 1.2) show a near 1∶1 match for experimentally measured results.

To investigate if this was indeed the case, we revised the model to include both VNG0702H and VNG2581H as independent elements. We first incorporated subtle differences in trafficking functions (Model 1.1, Figure 4A). Specifically, both metallochaperones were capable of directly binding intracellular Cu, distributing Cu to Q, activating VNG1179C by allocating excess Cu to the metalloregulator, and trafficking excess Cu to YvgX. However, only one metallochaperone was ideally suited for each task, while the other was 10-fold less efficient, resulting in “strong” and “weak” interactions, respectively.

In a revised version of this new model, we incorporated distinct functions for each metallochaperone (Model 1.2) (Figure 4B). In this model, Cu trafficking by VNG0702H is restricted to YvgX, while VNG2581H is responsible for Cu allocation to all remaining targets including VNG1179C and *Q*. Importantly, VNG2581H was modeled as the only metallochaperone that actively binds free intracellular Cu ions, thus production of Cu-bound VNG0702H requires a hand-off of Cu from VNG2581H, an inter-metallochaperone interaction that has been previously observed in eukaryotes [27]. Thus, Models 1.1 and 1.2 represent alternate extremes for functions of the two metallochaperones. Eliminating one of the two models would ascertain whether the two metallochaperones have interchangeable (Model 1.1) or distinct (Model 1.2) functions in intracellular Cu trafficking and buffering.

The two models made significantly different predictions of intracellular Cu levels at steady state under Cu stress. Model 1.1 predicted increased Cu levels in all single and double chaperone mutant backgrounds (overexpression and deletion), except *Δ0702Δ2581*, in which intracellular Cu was predicted to be at *wt* levels (Figure 4C). In contrast, Model 1.2 predicted increased Cu levels in all mutants except when only VNG0702H is overexpressed (Figure 4D).

#### Experimental verification

To discriminate between the two revised models, we performed additional ICP-MS analysis of strains in which the metallochaperones were deleted or overexpressed one at a time. These experiments revealed that both strains with single gene deletions (*Δ0702* and *Δ2581*) had elevated intracellular Cu levels similar to that of *Δ0702Δ2581* (Figure 4E). Interestingly, overexpression of VNG0702H (*Ω0702* – pLP18, Figure S7) and VNG2581H (*Ω2581* – pLP19, Figure S8) had drastically different consequences. Intracellular Cu concentration in *Ω0702* was lower than *wt*, while *Ω2581* accumulated Cu at levels similar to deletion strains. Based on these experimental data, the majority of Model 1.1 predictions were incorrect (Figure 4C). On the other hand, with the exception of intracellular Cu levels in *Ω0702Ω2581* case, all other predictions made using Model 1.2 were reasonably accurate (Figure 4D).

#### Steady-state analysis improves model accuracy

To better understand the discrepancies between Model 1.2 and experimental data, we turned to a model of the steady-state dependence of intracellular Cu level (*Cu_in_*) on the two metallochaperones (Model 2). We chose the generalized Hill function method [42], among others [43], [44], to construct Model 2 that is represented by the following rational polynomial:
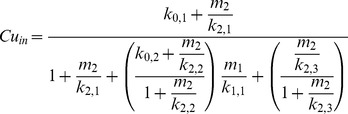
(25)where 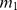 and 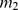 are the intracellular concentrations of the metallochaperones VNG0702H and VNG2581H, respectively. Using parameter values of:
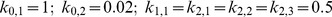
(26)for appropriate combinations of metallochaperone expression levels,
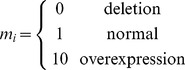
(27)this model was able to accurately recapitulate our steady state experimental observations (Figure S9A) with the unique and interpretable role of each parameter in eq. (25) (Figure S10).

Detailed inspection reveals that the cross term in the denominator of Model 2:
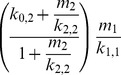
(28)validates the core regulatory network structure used in Model 1.2. Specifically, it is indicative of the dependence of Cu trafficking activity of VNG0702H on the activity of VNG2581H. In Model 1.2, this is represented as three interactions: Cu trafficking via VNG2581H to VNG1179C with subsequent transcriptional activation of VNG0702, and Cu trafficking between VNG0702H and VNG2581H.

In addition, parameters that allow Model 2 to accurately fit experimental data highlight key dynamics missing from Model 1.2. The constant 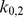 is required to produce decreased intracellular Cu in the *Δ2581* background versus *Δ0702Δ2581* and implies a basal ability of VNG0702H to directly bind Cu. Similarly, the constant 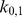 represents basal capacity of VNG2581H to traffick Cu to YvgX. Lastly, there are saturation dynamics for VNG2581H (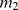) in the denominator:
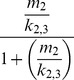
(29)and for VNG0702H (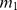) when VNG2581H is deleted (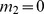):
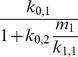
(30)which imply limited Cu binding capacity in each metallochaperone pool.

Basal activities represented by 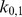 and 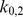 represented weak Cu trafficking interactions and were straightforward to add to Model 1.2. On the other hand, saturation of the binding capacities of both metallochaperones could stem from multiple mechanisms. First, there could be additional low-copy intermediates involved in relaying Cu from metallochaperones to downstream targets. Second, the metallochaperones themselves could exist at low copies despite active transcriptional production. To be as parsimonious as possible, the latter mechanism was investigated via explicit degradation of metallochaperone proteins on the order of the cell growth rate. In combination with the above modifications, the resultant dynamic model (Model 1.3, Figure S11, SBML version in supplement) was able to accurately predict experimentally observed intracellular Cu levels (Figures 4F and S9B).

Together, the revised model and experimental data support unique roles for the two metallochaperones in *H. salinarum* in a manner that is reminiscent of Cu trafficking in eukaryotes [27].

#### Sensitivity analysis

To further characterize Model 2, we performed a sensitivity analysis of steady-state copper concentration relative to changes in each of the underlying model parameters. To do this, we varied each parameter (holding all others constant) such that the log_10_ transform of the ratio between the final and initial parameter values P_f_/P_0_ was within [−1,1]. Results of this analysis are shown in Figure S12 and are interpreted as follows.

Consider the parameter k_1,1_, the activity constant for VNG0702H (species m_1_ in Model 2). As a validation, there should be no changes in the model performance for any strain with *Δ0702* (e.g. *Δ0702* and *Δ0702Δ2581*) as k_1,1_ is varied. This is observed as two white stripes in the first plot within Figure S12, as expected.

For all other strains there is a positive correlation between k_1,1_ and changes in steady-state copper levels. The steady-state level of intracellular Cu is affected by VNG0702H only through the cross term in the denominator of eq. (25). Increasing the value of k_1,1_ lowers the activity of VNG0702H (i.e. its ability to bind and transfer Cu ions to VNG0700G) which acts as a VNG2581H dependent inhibitor. Decreasing the inhibitory role of VNG0702H increases the steady-state level of intracellular Cu, hence a positive correlation. Importantly, such analysis shows the specific sensitivities of each chaperone expression states. Interestingly, strains in which VNG0702H is overexpressed are the most sensitive to changes to k_1,1_.

To further characterize Model 1.3, a sensitivity analysis as described above was performed. The results of this analysis are shown in Figure S13. As was the case for Model 2, reducing the activity of VNG0702H results in an increase in intracellular Cu. However, in Model 1.3 one finds that this reduction of activity can occur in several ways. First, the binding affinity of VNG0702H can be reduced (decreasing the equilibrium ratio between apo-/Cu-bound VNG0702H, or apo-VNG0702H/Cu-VNG2581H). Second, trafficking efficiency of VNG0702H to VNG0700G can be reduced. Third and finally, the lifetime of VNG0702H proteins can be reduced via increased specific degradation.

Interestingly, strains that overexpress either one or both metallochaperones have similar sensitivity profiles as the wild-type. Conversely, *Δ2581* strains (e.g. *Δ2581* and *Δ0702Δ2581*) are the most distant from wild-type. These sensitivity profiles bear a strong resemblance to the different YvgX expression profiles and ICP-MS based intracellular Cu measurements across strains.

## Discussion

We have demonstrated that, similar to eukaryotes, the two chaperones in *H. salinarum* have distinct roles in Cu trafficking. Remarkably, by simultaneously modeling these distinct functions and their interplay we were able to explain why mutations that either increase or decrease the abundance of metallochaperones result in elevated intracellular Cu levels.

### Mechanistic explanation for increased intracellular Cu in metallochaperone deletion mutants

In absence of metallochaperones, trafficking of Cu is completely disrupted. When subjected to Cu stress, intracellular Cu concentration increases and eventually overcomes diffusional limitations to activate VNG1179C and increase transcription of *yvgX*. Despite its increased levels, YvgX is unable to receive Cu efficiently to perform its efflux function. Consequently, Cu levels are perpetually increased and VNG1179C remains locked in an activated state to constitutively drive the expression of *yvgX*. Importantly, it is the disrupted trafficking of Cu to YvgX that is ultimately responsible for similar consequences in both the single and double metallochaperone deletion mutants.

### Mechanistic explanation for increased intracellular Cu in metallochaperone overexpressing mutants

At the other end of the spectrum, when abundance of metallochaperones is increased, activation and deactivation of VNG1179C proceeds normally, and as a result we do not observe significant differences in transcriptional dynamics of *yvgX*. However, intracellular Cu level rises because of the increased number of Cu-binding sites in the overexpressed metallochaperones. Importantly, we observe this only with overexpression of VNG2581H; as increased VNG0702H expression also increases Cu efflux by trafficking to YvgX.

Thus, the interplay between metallochaperones with distinct trafficking roles is critical for modulating transcriptional responsiveness and efficacy of Cu efflux. We have demonstrated that this system of interactions among metallochaperones and their targets sets an upper threshold for intracellular Cu levels. As a result, biological systems are under stringent selection pressure to maintain a fine balance in the activity of metallochaperones and their abundance. Changes to either can significantly affect responsiveness of the metalloregulator to modulate transcriptional dynamics of the efflux pump, and, ultimately, alter the homeostatic intracellular level of Cu.

**Figure 5:**
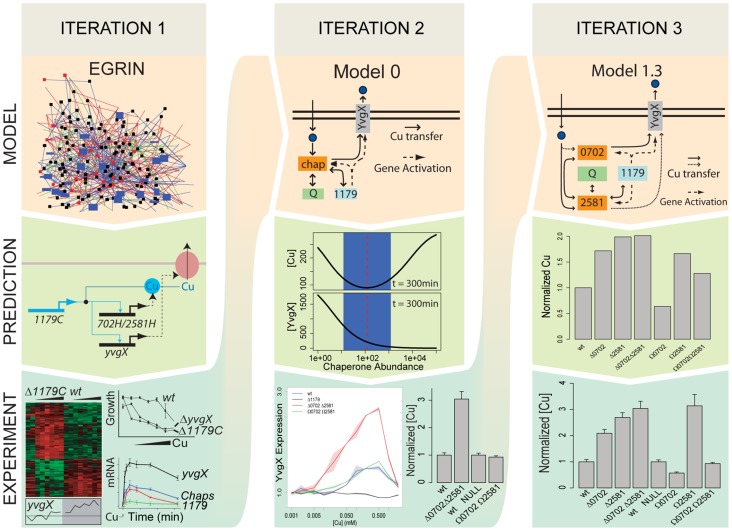
Three iterations of data-driven modeling, model prediction, and experimental testing has revealed novel insights into the role of metallochaperones in regulation of transcriptional dynamics of Cu-responsive efflux as well setting the homeostatic limits for intracellular Cu levels. In Iteration 1, a systems scale environmental and genetic regulatory influence network (EGRIN) model for transcriptional control of physiology aided in the discovery of components of the Cu efflux circuit in *H. salinarum*. The experimental validations of EGRIN predictions was instrumental in the formulation of Model 0, which predicted consequences of deleting or overexpressing metallochaperones on transcriptional dynamics of the efflux pump as well as the intracellular Cu concentration. In Iteration 2, the specific predictions of Model 0 were experimentally tested by assaying transcriptional dynamics of the efflux pump over a range of Cu concentrations, and also by simultaneously measuring intracellular Cu in chaperone deletion and overexpression mutants. While the most of Model 0 predictions were validated by these experiments, disparity in the Model 0 prediction and experimental data vis-à-vis intracellular Cu level in the overexpression mutant initiated the next iteration. In Iteration 3, Model 1.3 was constructed based on predictions from models 1.1 (identically functional chaperones) and 1.2 (distinct chaperone functions); and topological inference from Model 2 (steady state generalized hill function). Measurements of intracellular Cu levels with ICP-MS directly match predictions from Model 1.3.

In conclusion, while mathematical modeling of Cu trafficking has been performed previously [45], what is unique about this study is that it incorporated three iterations of experimentation and computation to refine model architecture and parameters (Figure 5). The modeling incorporated actual experimental measurements, recapitulated known dynamics, and predicted new dynamics, properties, and functions that were experimentally validated. Similarly, the experimental validations included microarray analysis to assay global transcriptional dynamics of the Cu response, GFP-based reporter assays to measure high resolution transcriptional dynamics of the Cu efflux pump, and ICP-MS measurements of intracellular Cu levels. This iterative computation and experimentation strongly supports a novel buffering role for metallochaperones to mechanistically explain the cause for elevated intracellular Cu levels and overexpression of the ATP7A efflux pump in cell lines harboring ATOX1 mutations [19]. Ultimately, we have presented a quantitative model that explicitly demonstrates the role of metallochaperones in regulating intracellular Cu, a contribution that is novel to the field of metal biology. Indeed, additional iterations of experimentation and computation are necessary to further refine this model and reveal new insights.

## Supporting Information

Figure S1 **Protein sequence analysis of** ***H. salinarum*** **metallochaperones.**
(TIF)

Figure S2 **Vector map for pLP17.** pr0700>GFP reporting with null overexpression.
(TIF)

Figure S3 **Vector map for pLP20.** pr0700>GFP reporting with VNG0702H and VNG2581H overexpression.
(TIF)

Figure S4 **GFP reporter assays recapitulate transcriptional response of** ***yvgX*** **to a challenge with growth sub-inhibitory (0.85 mM) CuSO4.** Relative changes in *yvgX* transcript were monitored by flow cytometry measurements of fluorescence changes over time in cells with normal (*wt*, blue), deficient (*Δ0702Δ2581*, red), and increased (*Ω0702Ω2581*, green) metallochaperone levels. As expected, no fluorescence was detected in *Δ1179* cells (black line) – the negative control. Solid lines are averages of two independent biological replicates. Shaded areas show the spread between replicates.
(TIF)

Figure S5 **Growth phenotypes for metallochaperone deletion mutants.** Maximum growth rate of Cu efflux mutants in response to Cu show no significant reduction in fitness between *Δura3* (*wt*) and metallochaperone deletion strains. Conversely, deletion of either *VNG1179C* or *yvgX* results in a dramatic loss of fitness.
(TIF)

Figure S6 **Experimental confirmation of VNG0702H and VNG2581H overexpression in recombinant strains.** Transcript levels were measured using qPCR and confirmed to be at least 3–4 fold higher than *wt* expression with and without copper induction.
(TIF)

Figure S7 **Vector map for pLP18.** pr0700>GFP reporting with VNG0702H overexpression.
(TIF)

Figure S8 **Vector map for pLP19.** pr0700>GFP reporting with VNG2581H overexpression.
(TIF)

Figure S9 **Model predictions of intracellular Cu dependence on metallochaperone abundance recapitulate experimental observations.** (A) Predictions from a steady state model of intracellular Cu dependence on metallochaperones (Model 2) recapitulates ICP-MS results. (B) Predictions from a refined ODE model (Model 1.3) mirrors steady state model predictions and recapitulates ICP-MS results.
(TIF)

Figure S10 **Parameter essentiality analysis of Model 2.** (A) Original model. (B) k11→∞. (C) k21→∞. (D) k22→∞. (E) k23→∞. (F) k01 = 0. (G) k02 = 0. Red arrows point on the strains where the perturbed models have qualitative discrepancies with the experimental data (Figure 4E).
(TIF)

Figure S11 **Network diagram for Model 1.3 biochemical reactions.** Green nodes of the graph represent dynamical variables of Model 1.3. For the complete list of graphical notations used in the diagram see the Systems Biology Graphical Notation documentation (http://www.sbgn.org). The model diagram is available in XML format at https://github.com/wleepang/CuEfflux/blob/master/CuEfflux.xml and can be also viewed and explored in CellDesigner (http://www.celldesigner.org/).
(TIF)

Figure S12 **Parameter sensitivity analysis of Model 2.** Parameters were varied over a [10^−1^, 10^1^] range from their originally chosen values, holding all other parameters constant. The response of Model 2 (intracellular Cu) is shown as a heat map (blue = decrease, red = increase) over all strain backgrounds. Columns that vary from blue to red (bottom to top) indicate positive correlations, and vice versa. White bands indicate insensitive parameter ranges.
(TIF)

Figure S13 **Parameter sensitivity analysis of Model 1.3.** Parameters were varied over a [10^−1^, 10^1^] range from their originally chosen values, holding all other parameters constant. Predicted intracellular Cu levels were used as a measure of the response of Model 1.3 to specific parameter changes. Correlation coefficients between the log_10_ ratio of the intracellular Cu response and the log_10_ ratio of variations in each parameter are plotted as a heat map with hierarchical clustering. Black regions indicate where data is not available because of lack of either over expression or deletion of metallochaperones.
(TIF)

Software S1 **CuEffluxModel1p3.xml.** Model 1.3 as described in the text as an SBML version 2 level 1 formatted file.
(XML)

Text S1 **Description of additional protocols used in this study.**
(DOCX)
