# Supplementary figures and images for: Metallochaperones Regulate Intracellular Copper Levels
Source: PLoS Comput Biol. 2013 Jan 17;9(1):e1002880. doi: 10.1371/journal.pcbi.1002880 (PMC3551603; doi:10.1371/journal.pcbi.1002880)

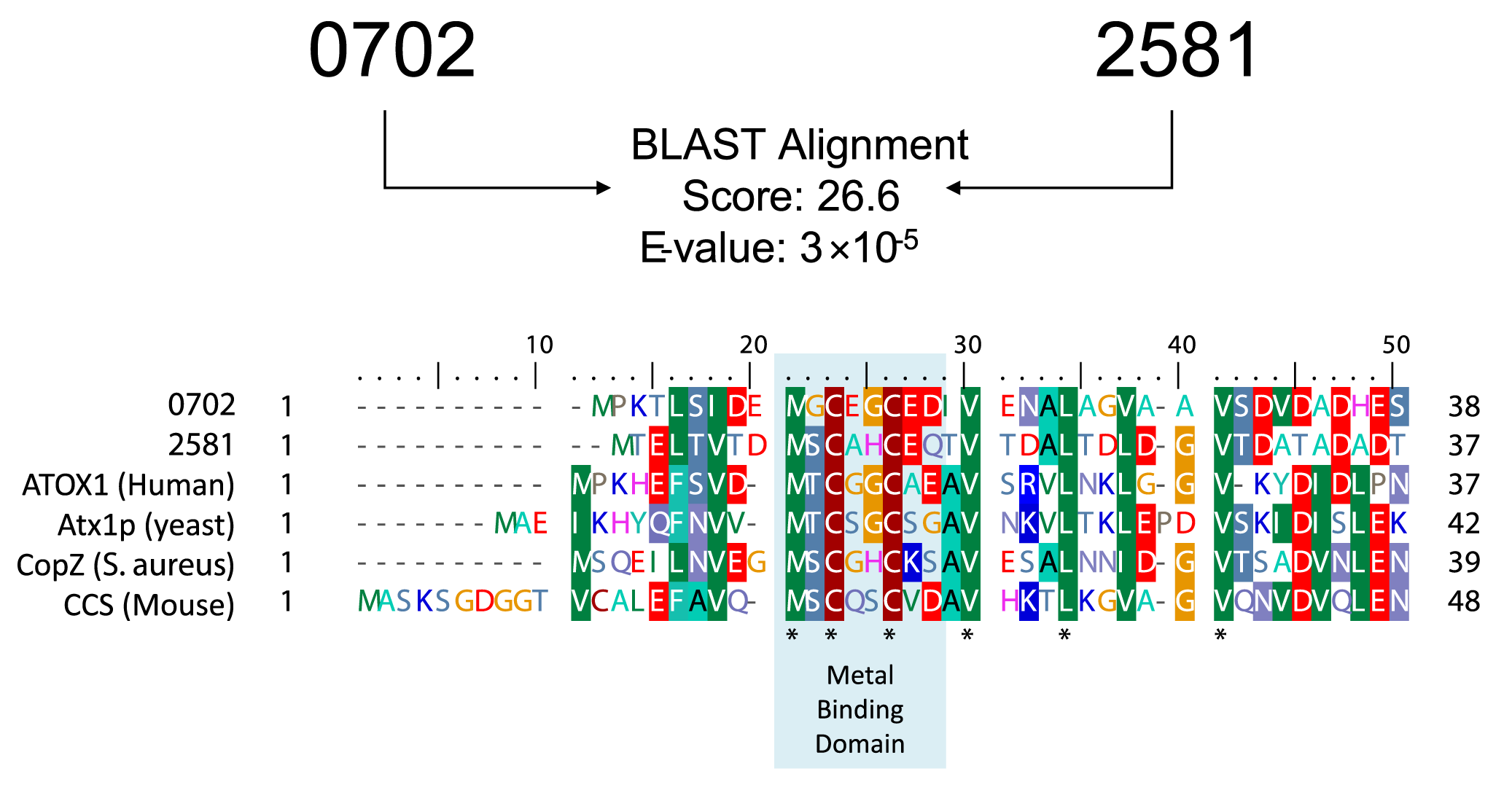

Supplement: Figure S1 — Protein sequence analysis of H. salinarum metallochaperones. (TIF) [file pcbi.1002880.s001.tif]

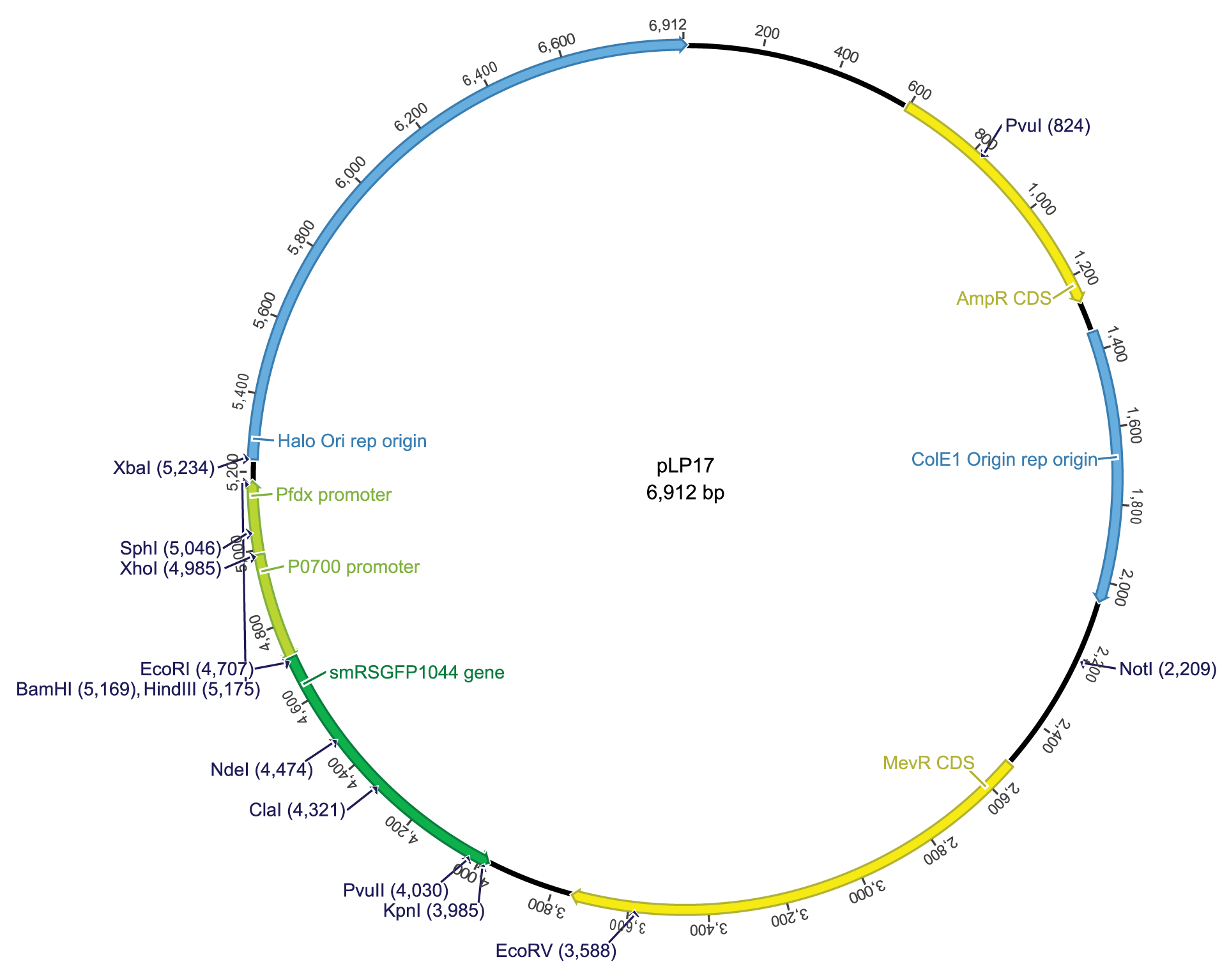

Supplement: Figure S2 — Vector map for pLP17. pr0700>GFP reporting with null overexpression. (TIF) [file pcbi.1002880.s002.tif]

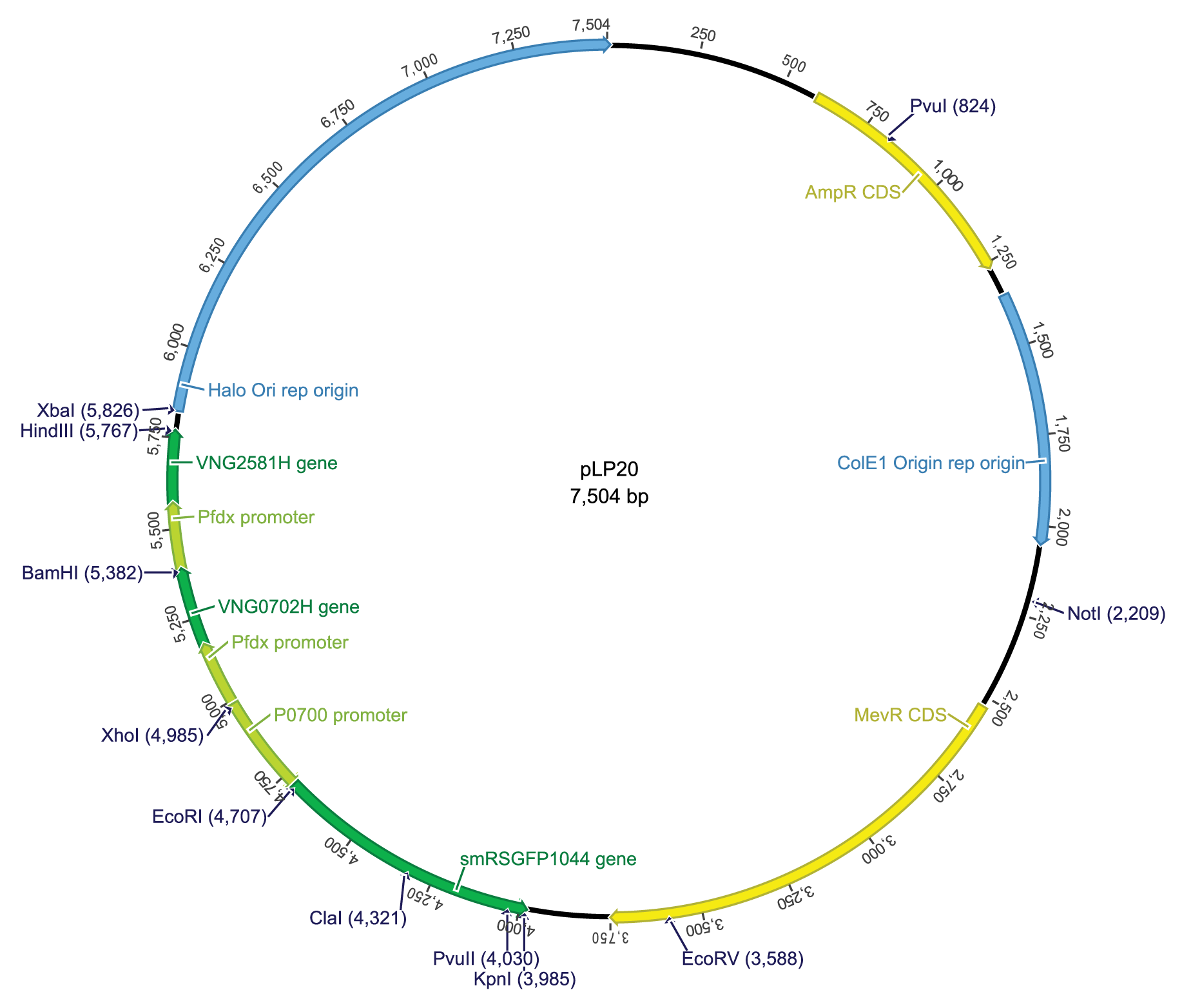

Supplement: Figure S3 — Vector map for pLP20. pr0700>GFP reporting with VNG0702H and VNG2581H overexpression. (TIF) [file pcbi.1002880.s003.tif]

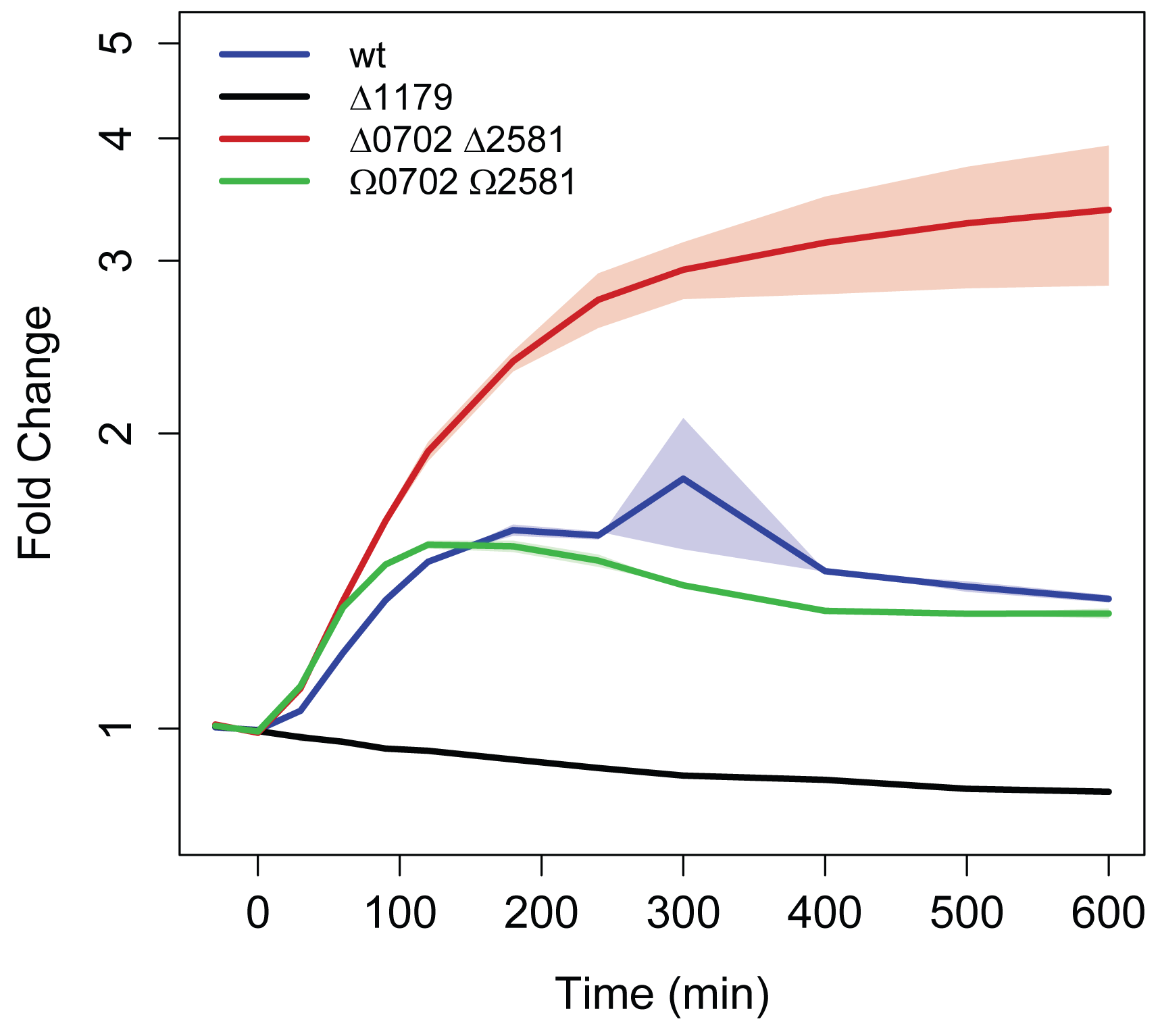

Supplement: Figure S4 — GFP reporter assays recapitulate transcriptional response of yvgX to a challenge with growth sub-inhibitory (0.85 mM) CuSO4. Relative changes in yvgX transcript were monitored by flow cytometry measurements of fluorescence changes over time in cells with normal (wt, blue), deficient (Δ0702Δ2581, red), and increased (Ω0702Ω2581, green) metallochaperone levels. As expected, no fluorescence was detected in Δ1179 cells (black line) – the negative control. Solid lines are averages of two independent biological replicates. Shaded areas show the spread between replicates. (TIF) [file pcbi.1002880.s004.tif]

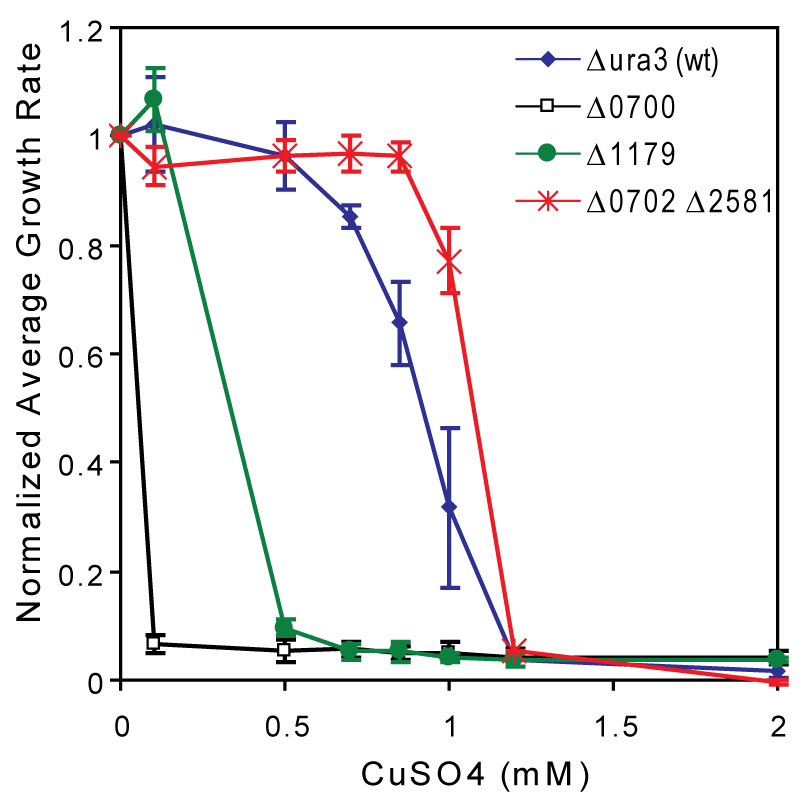

Supplement: Figure S5 — Growth phenotypes for metallochaperone deletion mutants. Maximum growth rate of Cu efflux mutants in response to Cu show no significant reduction in fitness between Δura3 (wt) and metallochaperone deletion strains. Conversely, deletion of either VNG1179C or yvgX results in a dramatic loss of fitness. (TIF) [file pcbi.1002880.s005.tif]

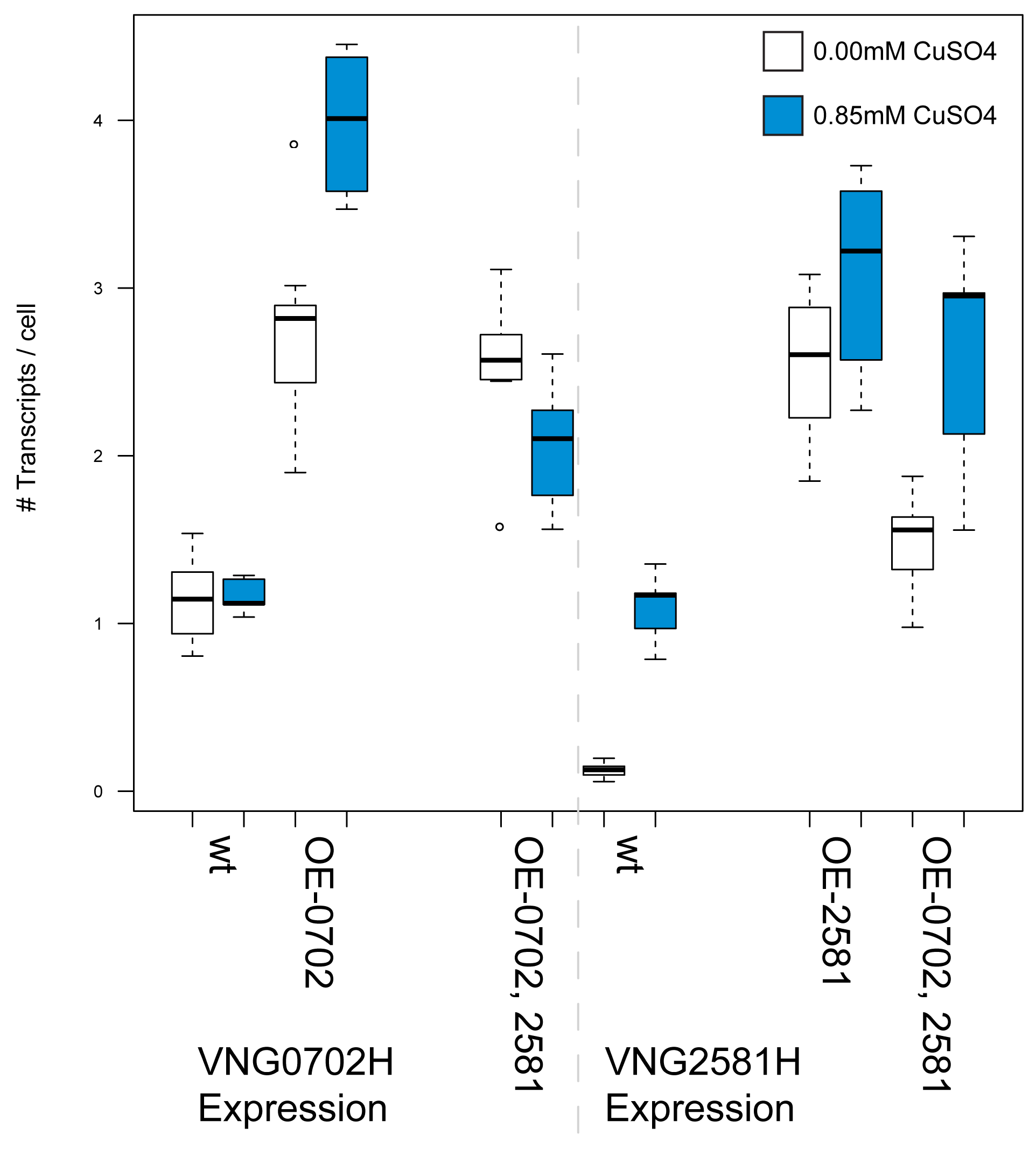

Supplement: Figure S6 — Experimental confirmation of VNG0702H and VNG2581H overexpression in recombinant strains. Transcript levels were measured using qPCR and confirmed to be at least 3–4 fold higher than wt expression with and without copper induction. (TIF) [file pcbi.1002880.s006.tif]

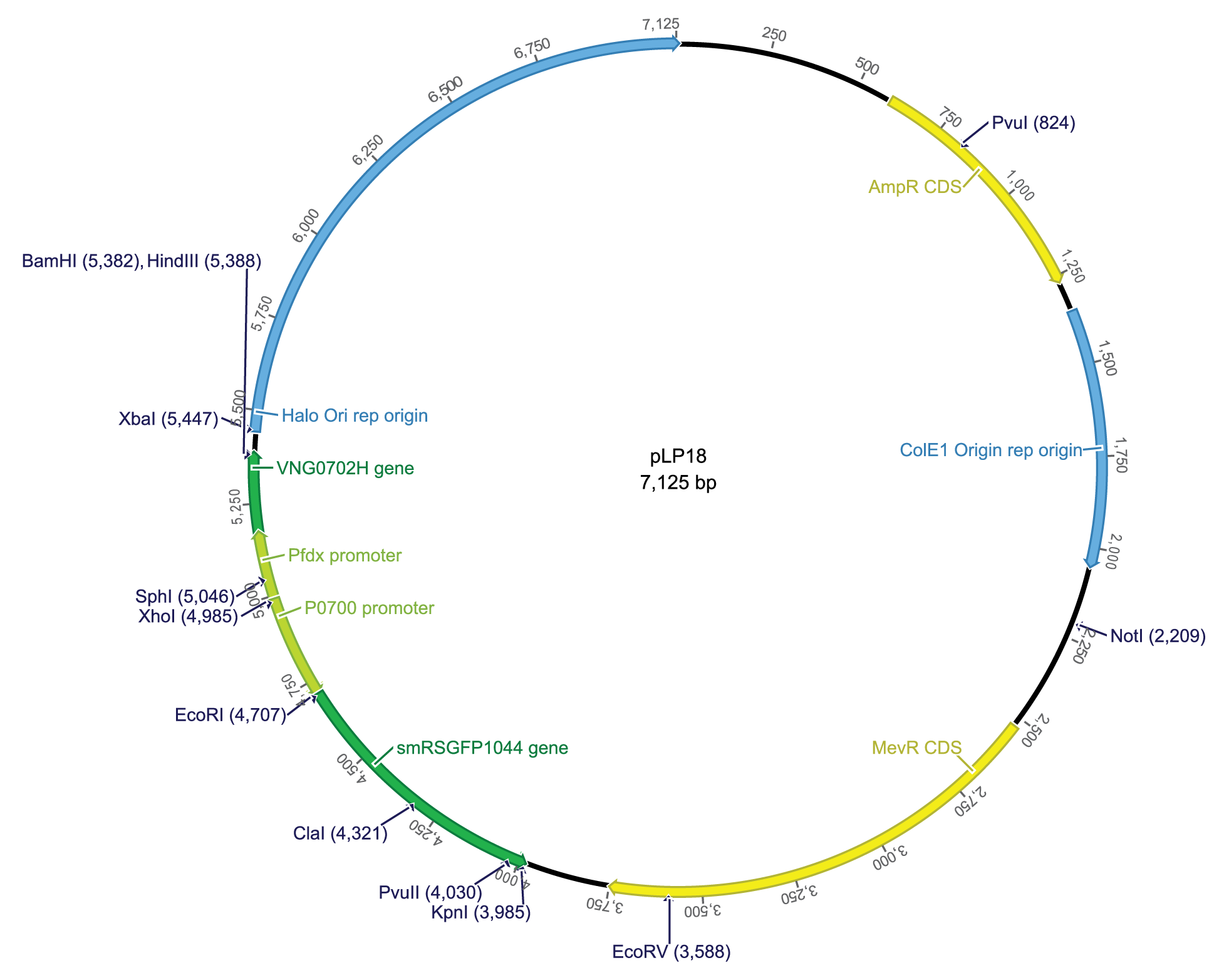

Supplement: Figure S7 — Vector map for pLP18. pr0700>GFP reporting with VNG0702H overexpression. (TIF) [file pcbi.1002880.s007.tif]

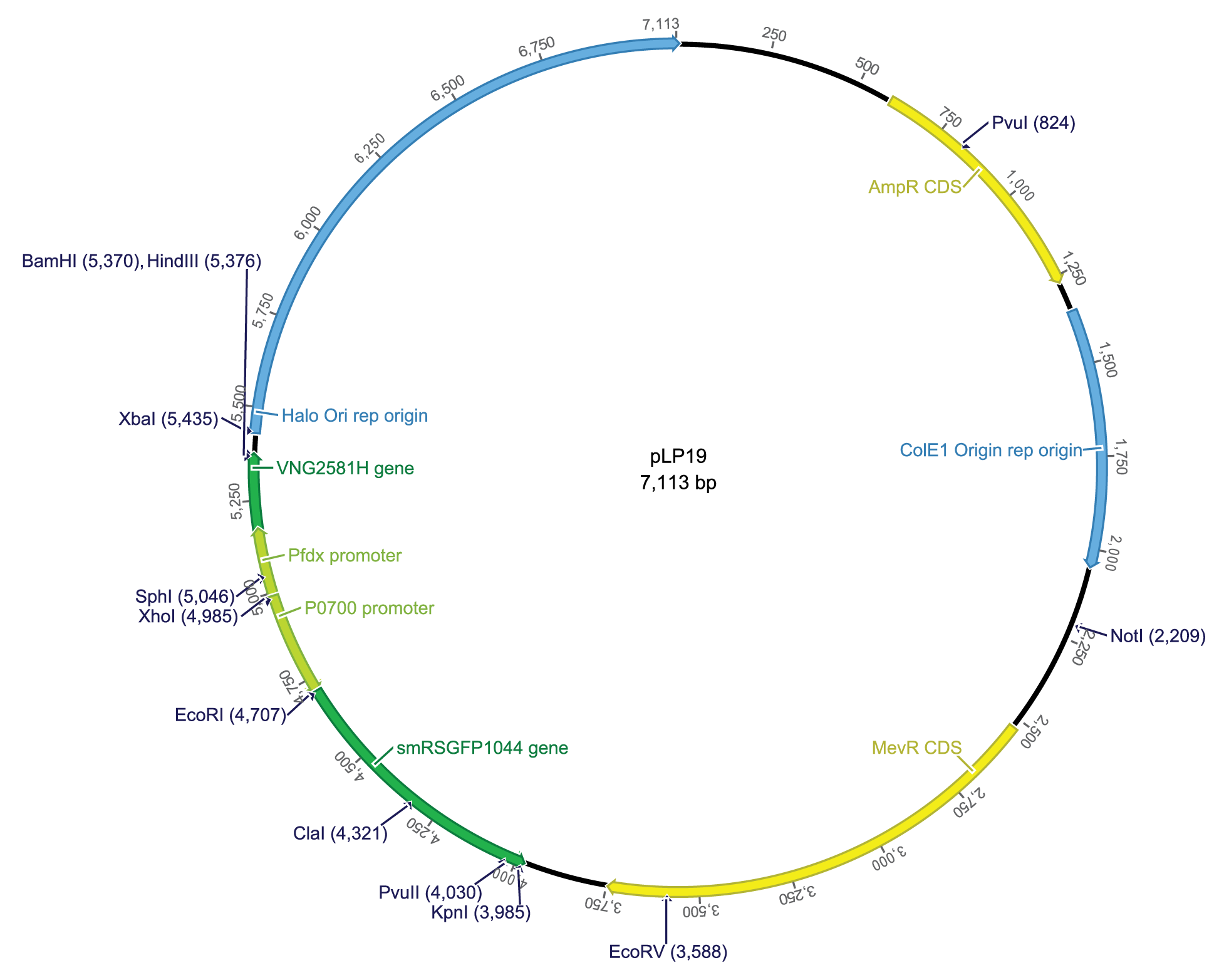

Supplement: Figure S8 — Vector map for pLP19. pr0700>GFP reporting with VNG2581H overexpression. (TIF) [file pcbi.1002880.s008.tif]

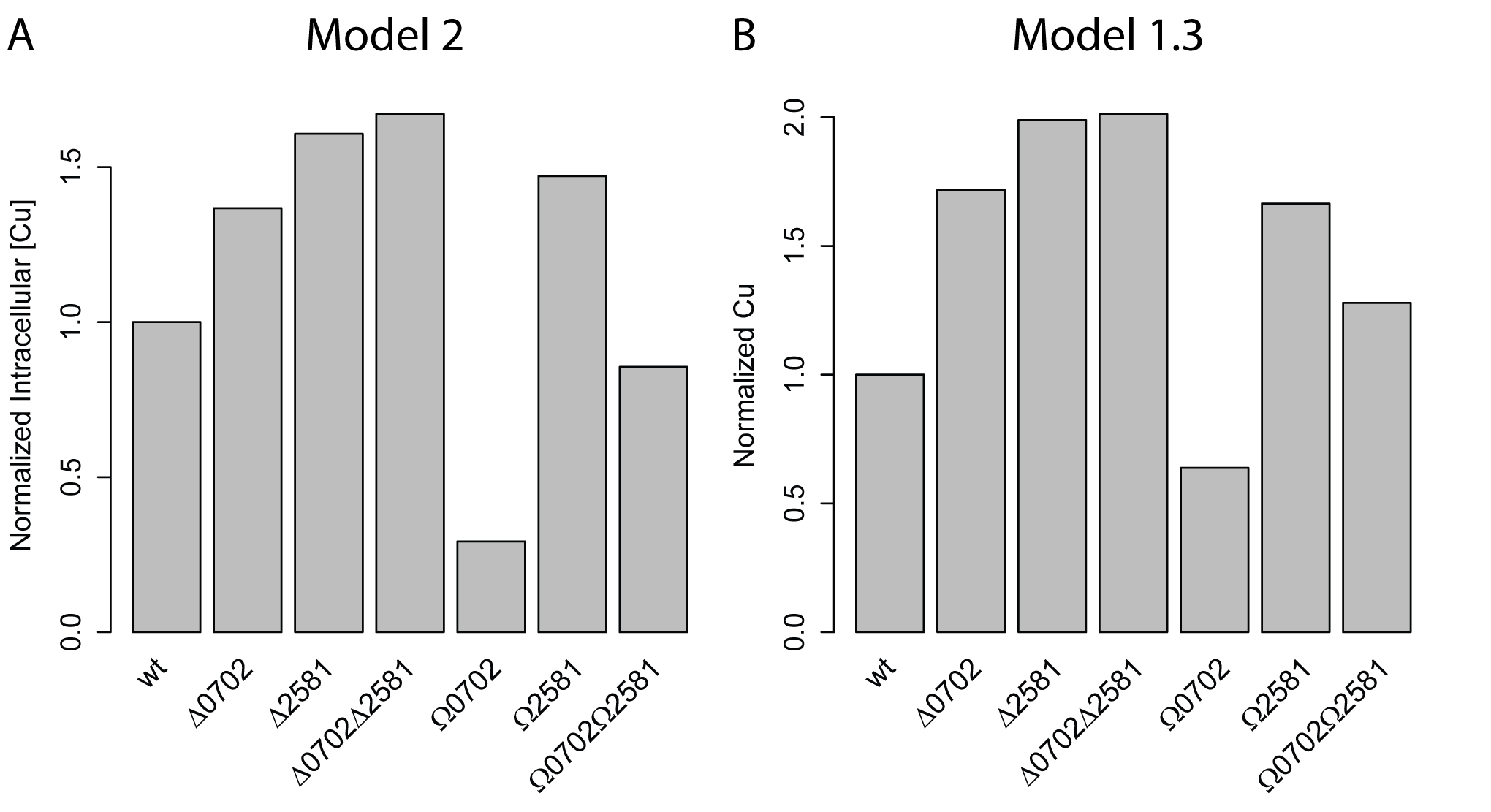

Supplement: Figure S9 — Model predictions of intracellular Cu dependence on metallochaperone abundance recapitulate experimental observations. (A) Predictions from a steady state model of intracellular Cu dependence on metallochaperones (Model 2) recapitulates ICP-MS results. (B) Predictions from a refined ODE model (Model 1.3) mirrors steady state model predictions and recapitulates ICP-MS results. (TIF) [file pcbi.1002880.s009.tif]

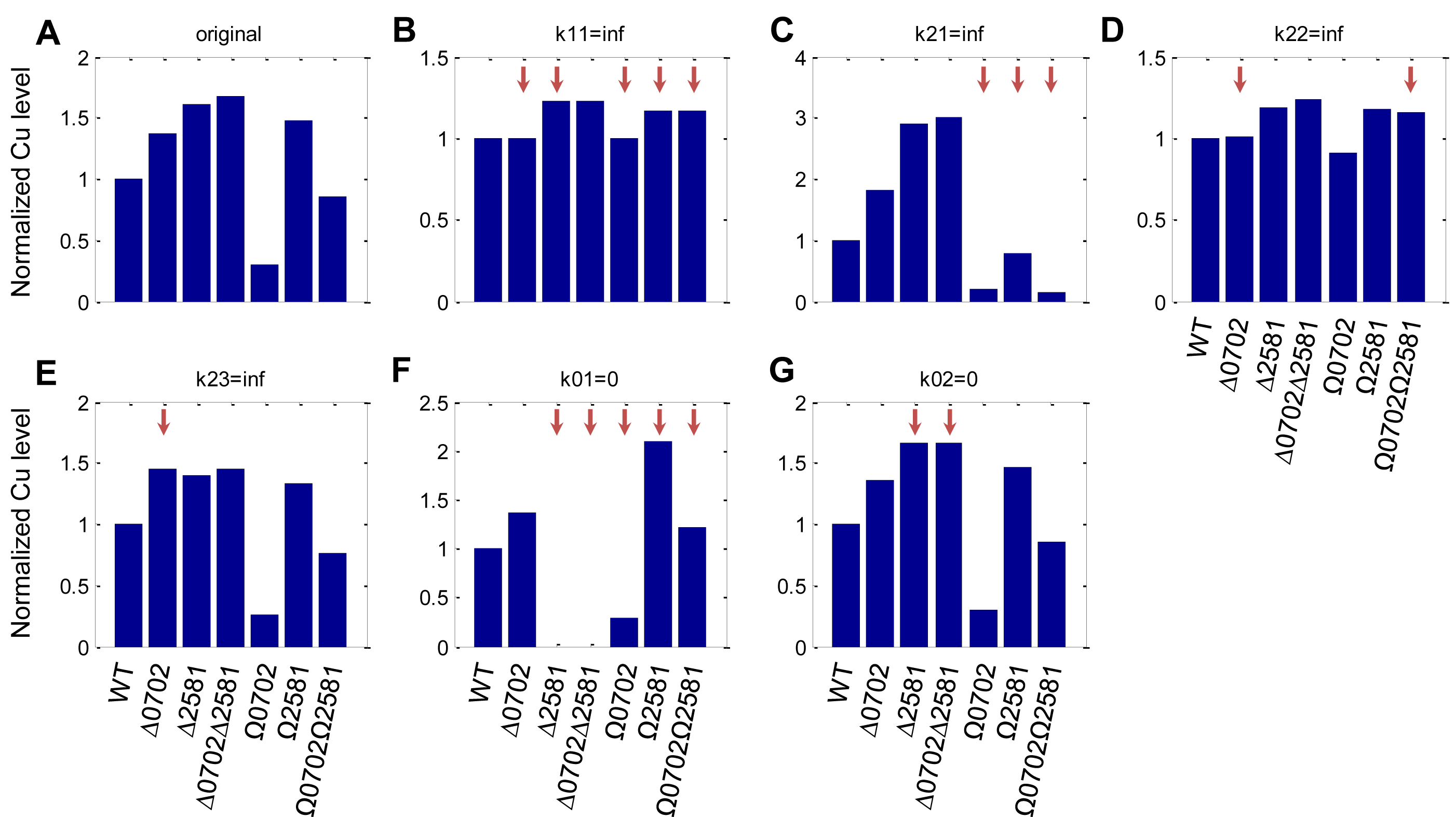

Supplement: Figure S10 — Parameter essentiality analysis of Model 2. (A) Original model. (B) k11→∞. (C) k21→∞. (D) k22→∞. (E) k23→∞. (F) k01 = 0. (G) k02 = 0. Red arrows point on the strains where the perturbed models have qualitative discrepancies with the experimental data (Figure 4E). (TIF) [file pcbi.1002880.s010.tif]

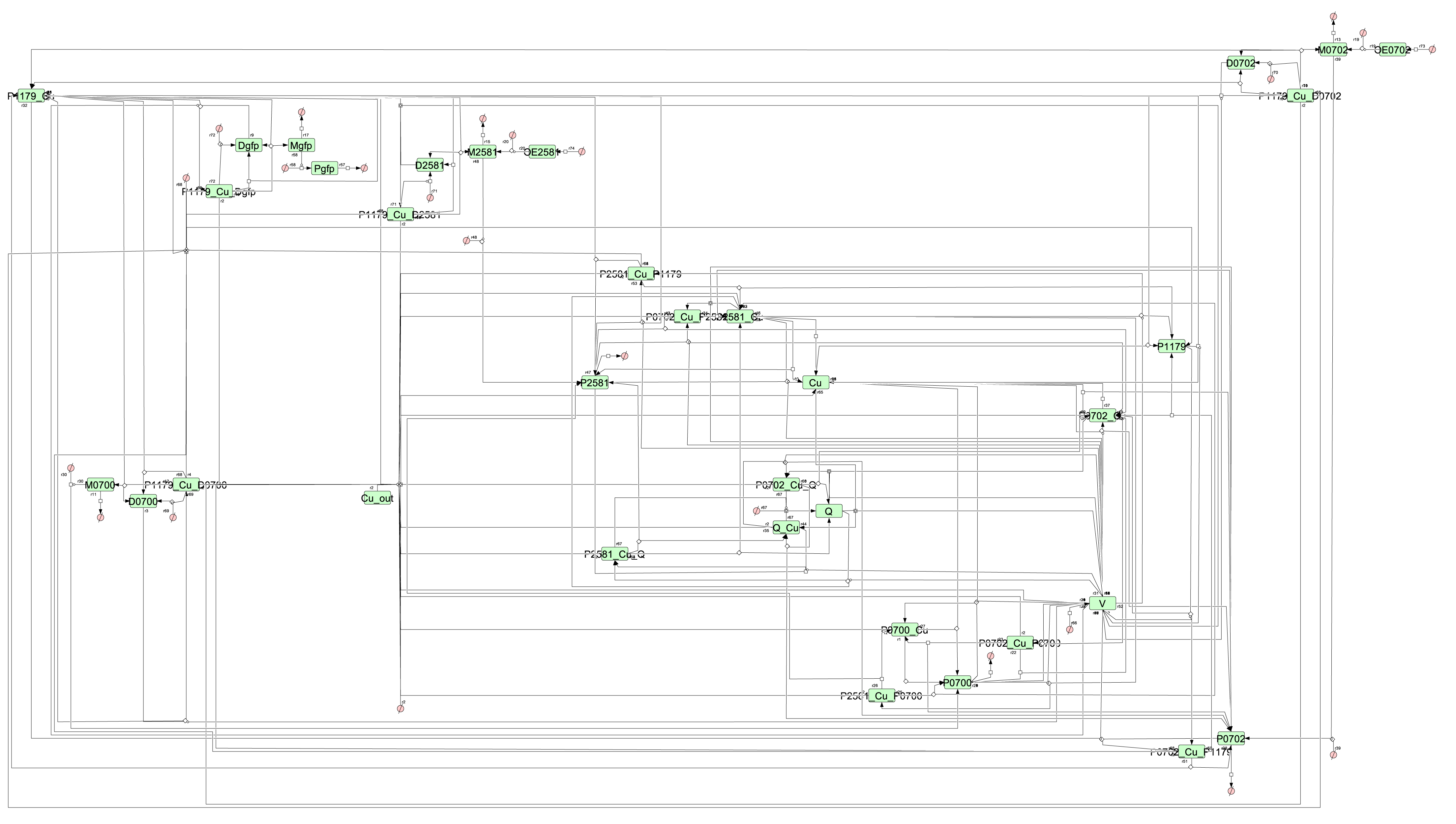

Supplement: Figure S11 — Network diagram for Model 1.3 biochemical reactions. Green nodes of the graph represent dynamical variables of Model 1.3. For the complete list of graphical notations used in the diagram see the Systems Biology Graphical Notation documentation (http://www.sbgn.org). The model diagram is available in XML format at https://github.com/wleepang/CuEfflux/blob/master/CuEfflux.xml and can be also viewed and explored in CellDesigner (http://www.celldesigner.org/). (TIF) [file pcbi.1002880.s011.tif]

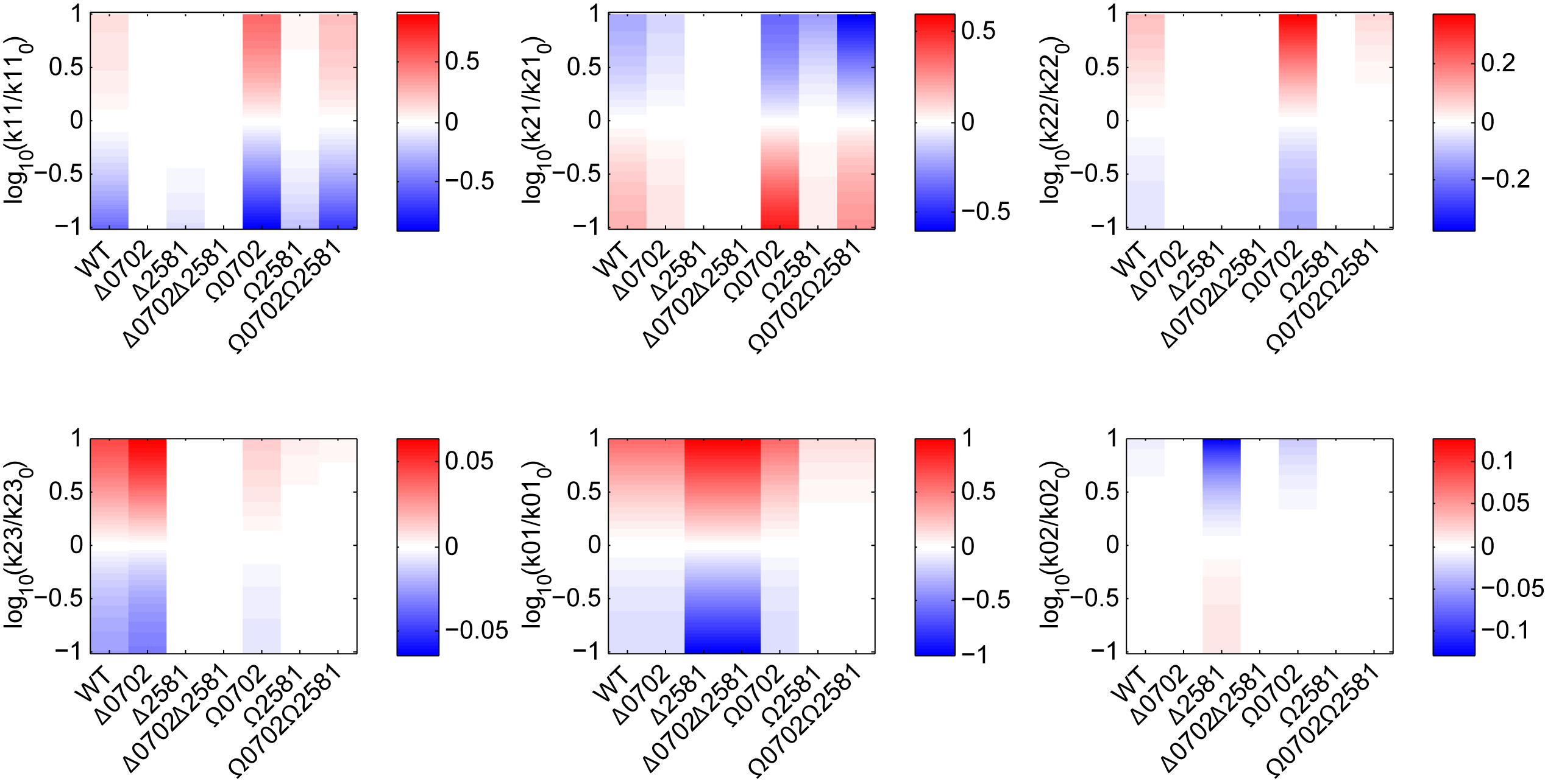

Supplement: Figure S12 — Parameter sensitivity analysis of Model 2. Parameters were varied over a [10−1, 101] range from their originally chosen values, holding all other parameters constant. The response of Model 2 (intracellular Cu) is shown as a heat map (blue = decrease, red = increase) over all strain backgrounds. Columns that vary from blue to red (bottom to top) indicate positive correlations, and vice versa. White bands indicate insensitive parameter ranges. (TIF) [file pcbi.1002880.s012.tif]

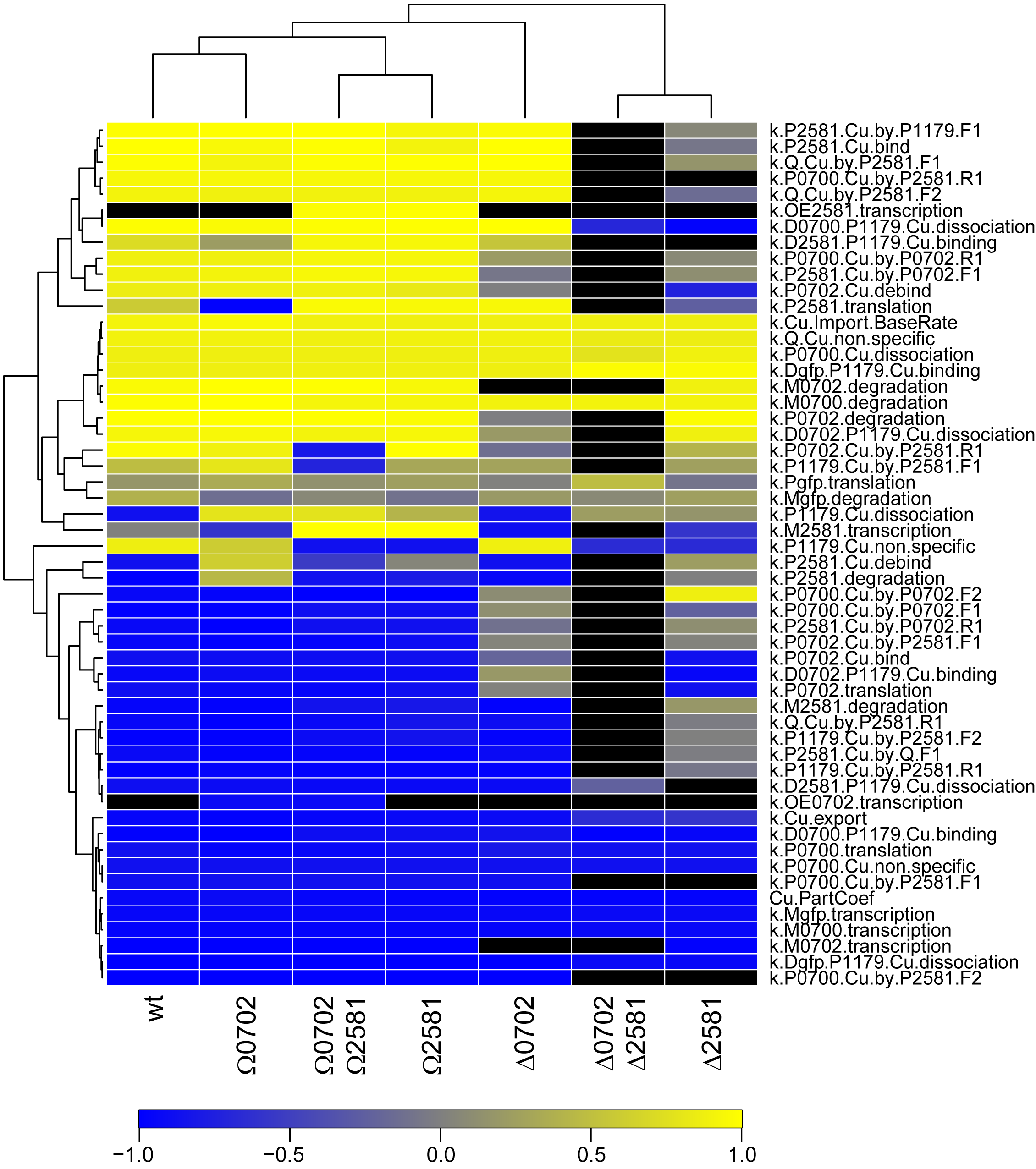

Supplement: Figure S13 — Parameter sensitivity analysis of Model 1.3. Parameters were varied over a [10−1, 101] range from their originally chosen values, holding all other parameters constant. Predicted intracellular Cu levels were used as a measure of the response of Model 1.3 to specific parameter changes. Correlation coefficients between the log10 ratio of the intracellular Cu response and the log10 ratio of variations in each parameter are plotted as a heat map with hierarchical clustering. Black regions indicate where data is not available because of lack of either over expression or deletion of metallochaperones. (TIF) [file pcbi.1002880.s013.tif]
