# Supplementary material for: Metallochaperones Regulate Intracellular Copper Levels
Source: PLoS Comput Biol. 2013 Jan 17;9(1):e1002880. doi: 10.1371/journal.pcbi.1002880 (PMC3551603; doi:10.1371/journal.pcbi.1002880)
Supplement: Text S1 — Description of additional protocols used in this study. (DOCX) [file pcbi.1002880.s015.docx]

# Metallochaperones regulate intracellular Copper levels

W. Lee Pang^1,7^, Amardeep Kaur^1^, Alexander V. Ratushny^1,2^, Aleksandar Cvetkovic^3,6^, Sunil Kumar^3^, Min Pan^1^, Adam P. Arkin^4^, John D. Aitchison^1,2^, Michael W. W. Adams^3^, and Nitin S. Baliga^1,5,*^

1. Institute for Systems Biology, Seattle, WA
2. Seattle Biomedical Research Institute, Seattle, WA
3. Department of Biochemistry and Molecular Biology, University of Georgia, Athens, GA
4. Department of Bioengineering, University of California, Berkeley, CA
5. Department of Microbiology, University of Washington, Seattle, WA
6. Currently: Pall Life Sciences, Boston, MA
7. Currently: Genomatica, Inc., San Diego, CA

To whom correspondence should be addressed: Nitin S. Baliga, Institute for Systems Biology, 401 Terry Ave N., Seattle WA, 98109. Tel.: (206) 732-1266. Fax: (206) 732-1299. E-mail: Nitin.Baliga@systemsbiology.org.

## Supplemental Data

[ICP-MS Control Samples 1](#_Toc316989396)

[Metallochaperone deletion phenotypes 1](#_Toc316989397)

[Expression vector construction 2](#_Toc316989399)

[Quantitative PCR protocol 2](#_Toc316989400)

[References 3](#_Toc316989401)

### ICP-MS Control Samples

| Description | Average  (uM) | Std.Dev  (uM) | Replicates |
| --- | --- | --- | --- |
| MilliQ water (sample tube wash) | 0.161 | 0.174 | 3 |
| MilliQ water, metal free | 0.003 | 0.004 | 2 |
| Basal Salts Solution (BSS), metal free | 0.346 | 0.071 | 3 |
| Complete Medium (CM) | 0.530 | NA | 1 |
| CM + Uracil | 0.689 | 0.045 | 2 |
| CM + Mevinolin + Uracil | 1.841 | 2.013 | 3 |

**Table S1**. Cu abundance in ICP-MS control samples

### Metallochaperone deletion phenotypes

We assayed metallochaperone deletion mutants for growth under varying concentrations of Cu. Unlike knockouts for *VNG1179C* or *yvgX*, metallochaperone knockout mutants exhibited no phenotypic defects (Figure S5).

### Expression vector construction

The promoter for VNG0700G (yvgX), cloned directly from the *H. salinarum* genome, is the intergenic space upstream of and including the predicted protein start codon (approx. 200bp). GFP (smRSGFP1044) was cloned from pJAM1044 [[1](#_ENREF_1)] . Since transcript termination signals have yet to be identified in *H. salinarum*, the pr0700>smRSGFP1044 fusion is divergent of ferrodoxin promoter (prFdx) fusions to avoid aberrant overexpression of GFP. The genes for VNG0702H and VNG2581H were each cloned from the *H. salinarum* genome according to predicted ORF boundaries. Overexpression was confirmed using qPCR (Figure S6).

### Quantitative PCR protocol

Total RNA was extracted with mirVana™ miRNA Isolation Kit (Ambion – AM1561) and treated with DNase (DNA-free kit-AM1561) to remove genomic DNA contamination. The integrity of the RNA samples was checked using an Agilent Bioanalyzer. Power SYBR® Green RNA-to-CT ™ 1-Step Kit (Applied Biosystems – 4389986) was used to prepare one-step RT-PCR reaction mix with SYBR® Green reagents. Reactions were cycled and measured on a 7900HT from Applied Biosystems in 384-well plate format. Absolute transcript levels were quantified using a standard curve and gene-specific primers for VNG0702H and VNG2581H.

### References

1. Reuter CJ, Maupin-Furlow JA (2004) Analysis of proteasome-dependent proteolysis in Haloferax volcanii cells, using short-lived green fluorescent proteins. Applied and environmental microbiology 70: 7530-7538.
